# Atomic-scale defect-mediated interfacial bonding in cold spray: FCC vs. BCC metal systems

**DOI:** 10.1016/j.apsadv.2026.101035

**Published:** 2026-09

**Authors:** Arash Kardani, Sara Bagherifard

**Affiliations:** Department of Mechanical Engineering, Politecnico di Milano, Milan, Italy

**Keywords:** Solid-state additive manufacturing, Interfacial bonding, Severe plastic deformation, Molecular dynamics simulation

## Abstract

The intense plastic deformation induced by the supersonic impact of powder particles during cold spray deposition determines particle-substrate interfacial properties. Herein, large-scale molecular dynamics simulations are employed to investigate the atomistic microstructural evolution of similar particle–substrate systems during impact. The focus is placed on elucidating the distinct responses exhibited by a FCC metal system (such as Al-Al) versus a BCC metal system (such as Fe-Fe) to enable a systematic comparison of deformation mechanisms, defect generation, and phase transformations intrinsic to each material type. Results indicate that the FCC system accommodates deformation through transient solid-state phase transformations from FCC to BCC and FCC to HCP, accompanied by localized interfacial rim melting and amorphization, as well as intense dislocation activity, which collectively enhance atomic intermixing and bonding. Conversely, deformation in the BCC system primarily occurs through atomic disordering and extensive twinning. At intermediate impact duration, the FCC system exhibits a significantly higher dislocation density, predominantly composed of partial dislocations. Interactions between these dislocations form sessile junctions that restrict slip and stabilize the interface. While, the BCC system is characterized by perfect dislocations closely associated with twin growth and thickening, which accommodate plastic deformation while locally limiting slip. In the final stage, both systems undergo grain refinement/recrystallization, forming new grains predominantly within the particle. Grain refinement in both systems increases grain boundary density, thereby strengthening interfacial bonding. Through these analyses, this study advances the understanding of fundamental bonding phenomena and the influence of intrinsic material properties on interfacial characteristics under solid-state deposition conditions.

## 1 Introduction

Among the evolving Additive Manufacturing (AM) techniques, Cold Spray (CS) stands out as an emergent technique distinguishing itself for its exceptional deposition rate, deposition efficiency, no size limits and unprecedented flexibility in material selection [[1], [2], [3], [4]]. This technique is widely used for producing corrosion- or wear-resistant coatings, repairing damaged components, and more recently for AM [5,6]. In CS, particles are accelerated by passing through a converging-diverging nozzle to supersonic speeds; as the particles collide with a substrate, they bond to it resulting in a coating formation. Specifically, the impingement and bonding occur under solid-state conditions, ensuring a deposition free from melting defects, with minimal oxide content and undesired residual stresses. Considering the advantages of the CS technology, considerable effort has been dedicated to investigations on various materials, mechanical and functional aspects of the process including optimizing CS parameters for improved bonding, expanding the range of materials used, and addressing key technological challenges associated with this technique, to name a few.

These studies have greatly advanced our knowledge regarding various aspects of CS process optimization and characterization; however, there remains a notable gap in understanding the fundamental metallurgical mechanisms involved in impact-induced bonding, specifically focusing on the relative mechanical and microstructural properties of the particle and the substrate.

Severe plastic deformation is a key factor in metallic particle-substrate metallurgical bonding [7,8]; therefore, intrinsic material characteristics, such as crystal lattice structure, crystallographic defects (e.g., dislocations and Stacking Faults (SFs)) as well as twinning, significantly influence the interfacial bonding characteristics. Since microstructural transformations arising from the nucleation and evolution of crystal defects occur at the atomic scale, atomic-level analyses such as Molecular Dynamics (MD) simulations are expected to provide valuable insight into the fundamental mechanisms governing bonding phenomena in CS. MD simulations have been implemented to study different aspects in CS. For instance, Rahmati et al. [9] examined how interfacial dislocation networks develop at the Cu particle-substrate contact and contribute to deformation during impact, thereby promoting metallurgical bonding. The significant role of dislocation network was also demonstrated experimentally by Lucas et al. [10], who investigated the impact of a single Cu particle on Cu substrates. Interestingly, their experimental results showed that particles required a lower impact velocity to bond to a work-hardened, cold-rolled substrate than to an annealed substrate, indicating that the higher dislocation density in the harder work-hardened substrate facilitated plastic deformation in the impacting particle [10]. This suggests that multiple, potentially coexisting mechanisms may govern the bonding process. For similar metallic systems with deformable crystal structures, low elastic stiffness (i.e., low Young’s modulus), and medium to low stacking fault energy (SFE), such as Cu and Al, it is therefore expected that the crystallographic orientations of both the particle and the substrate become particularly important, as they directly affect dislocation nucleation and evolution. Nevertheless, simulating the impact of Cu particles on Cu substrates demonstrated that difference in the crystallographic orientation of the particle had no significant effect on the interfacial dislocation density [11]. However, the distribution of dislocations significantly affected the particle's deformation and resulting morphology. Dislocation lattice along the crystallographic orientation of [110] was found to be concentrated near the particle’s center, and away from the interface. While, when the particle impacted along the [100] crystallographic direction, the largest contact area with the substrate was created, a phenomenon attributed to the concentration of dislocations at the interface. As such, these studies collectively suggest that particle-substrate bonding is often associated with the nucleation, interaction, and accumulation of a high dislocation density within the interfacial region. However, this trend should not be regarded as a universally applicable rule across all materials. For example, Li et al. [12] reported that, in a similar particle-substrate system of tungsten (W) under high velocity CS impact conditions, deformation twinning rather than dislocation activity became the dominant mechanism governing interfacial bonding.

Beyond the role of dislocations in interfacial plasticity of similar systems, solid-state phase transformation from crystalline to amorphous structure may also critically influence particle–substrate bonding. In this context, MD simulations have indicated that during the impact of Cu particle onto Cu substrate (under the assumption of weak interaction between the surface atoms of the particle and the substrate due to the existence of a brittle oxide layer), an increase in particle diameter led to more pronounced jetting at the particle's edges [13]. This increase was reported to arise from the greater likelihood that, for larger particles, the interfacial temperature may reach Cu’s melting point during impact. Formation of local melting and the extremely high cooling rate led to the development of an amorphous structure. It was also emphasized that a greater portion of atoms from the particle and the substrate became amorphous in the rim regions, thus, promoting the merging of the particle into the substrate [13]. These findings were similarly reported for the same CS system by Reddy et al., assuming no oxide layer at the particle–substrate interface [14]. As such, in addition to dislocations, amorphization may also play a prominent role in the severe plastic deformation of the interfacial region.

Recent MD studies [9,15,16], have revealed that the impact-induced severe plastic deformation occurs not only at the particle-substrate interface but also in regions distant from it; this extended microstructural evolution can promote stronger particle-substrate bonding. Grain refinement and recrystallization are vital factors affecting the particle-substrate bonding state and generally take place in regions away from the interface in both the particle and the substrate [17]. Mesoscale modeling using quasi-coarse-grained dynamics (QCGD) simulations of the CS deposition of Al-Al particle-substrate similar material system, showed that increased impact velocity leads to a greater number of grains forming in the regions adjacent to the interface [18]. These have been attributed to stress-driven Grain Boundary (GB) mobility and recrystallization at the particle-substrate interface. These evidences suggest that the formation of nanograins in regions adjacent to the interface, within both the particle and the substrate, can enhance the strength of this interfacial region [20,19].

A review of recent MD studies in this field shows that, despite extensive discussion of crystalline defects, phase transformations, interfacial microstructure, and severe plastic deformation, significant limitations remain. These research gaps can be categorized as follows: (i) The existing literature primarily focuses on metals with medium SFE and low elastic stiffness, particularly Cu. This emphasis likely arises from the extensive experimental database on CS deposition of Cu, positioning it as a suitable benchmark material for simulation-based research. However, this becomes a notable limitation in the atomic-level understanding of CS bonding and deformation mechanisms, particularly concerning metal’s crystal structures and mechanical properties; (ii) The details of how dislocations interact are still not fully understood. Earlier research did not address the specific types of dislocations or how they interact at and beyond the interface, leaving important gaps in knowledge. In particular, locked dislocations can have a major impact on the density of dislocations at the interface; (iii) Most phase transformations discussed in the literature are confined to the formation of melt or amorphous structures, paying less attention to crystalline phase transformations in pure metals under conditions of severe high strain-rate deformation [[21], [22], [23]], and thus leaving its potential effects on metallurgical bonding unexamined. (iv) FCC metals exhibit different deformation behaviors at atomic scale compared to BCC counterparts. In the MD simulations of CS, the metallurgical bonding mechanisms and the phenomena influencing severe plastic deformation in highly relevant BCC metals such as Fe remain insufficiently understood at the atomic scale. Thus, this study aims to address these research gaps by employing MD simulations to analyze the role of material structures, considering similar particle-substrate systems, in CS bonding. The main novelty of this study lies in moving beyond more extensively studied mechanisms such as oxide layer disruption, jetting, and mechanical interlocking toward the less explored atomic scale phenomena upon supersonic impact of particles to the substrate. Specifically, this work systematically investigates interfacial melting, amorphization, and solid-state phase transformations under high strain rate deformation, providing new insights into their roles in bonding and microstructural evolution as a function of material’s crystal structure. In this regard, two distinct material systems of FCC (Al-Al) and BCC (Fe-Fe) metals are considered to study microstructural changes resulting from severe plastic deformation during CS deposition of particles with a similar nature as the substrate. One of the main reasons for selecting these materials is their tendency to undergo phase transformations, amorphization, and their distinct SFEs compared to more studied metals such as Cu [21,24]. Following this introduction, Section 2 describes the simulation setup and analytical tools used. Section 3 presents the results, and Section 4 summarizes the main findings of this study.

## 2 Methodology and simulation setup

**Fig. 1:**
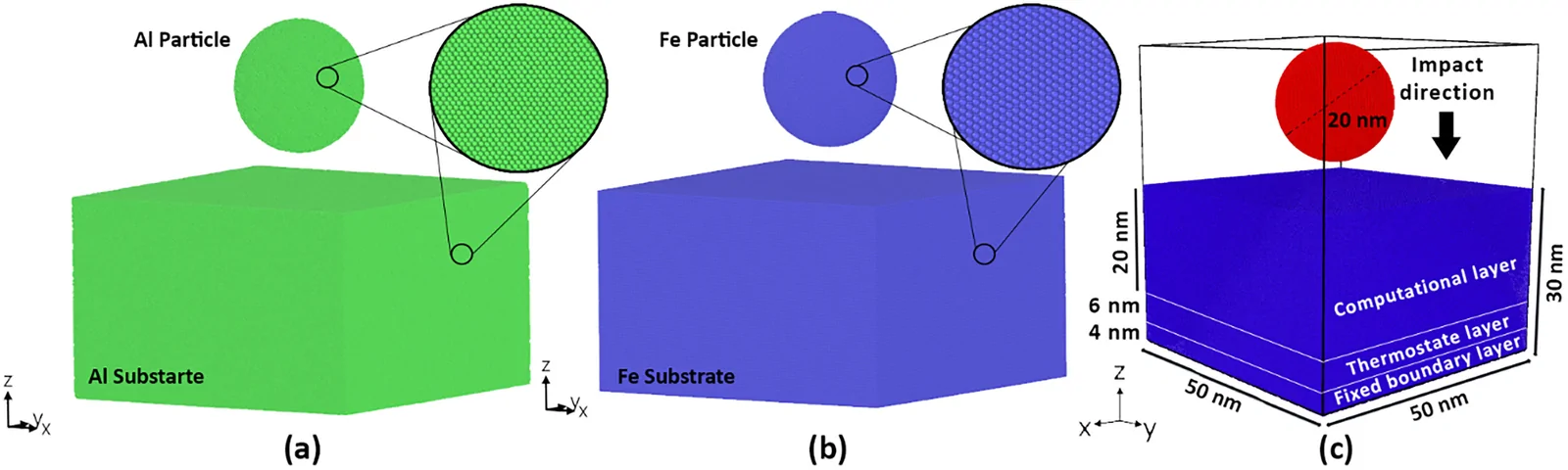
Initial configuration of the models: (a) FCC (Al-Al) system, (b) BCC (Fe-Fe) system, and (c) schematic illustration of the model representing its dimensions.

Figs. 1a and 1b show the initial configuration of the FCC (Al-Al) and the BCC (Fe-Fe) systems. For each model, the Representative Volume Element (RVE) comprised of a substrate measuring 50 × 50 × 30 nm^3^ and a particle with a 20 nm diameter. Although the particle size used in this model is smaller than that of actual particles to reduce computational cost, previous MD studies have demonstrated that this approach effectively replicates the principal mechanisms observed at both nano- and microscale levels [26,25,15]. In the Al system, which features an FCC structure with a lattice parameter of 4.04 Å, the system contained 4835,068 atoms, while in the Fe system, which has a BCC structure and a lattice parameter of 2.86 Å, the number of atoms was 8100,196. Although both models were the same size, the number of atoms differed because of the distinct atomic radii and lattice parameters of the two metals. The crystal orientations of both the substrate and the particle were aligned along the [100] direction in both models. While crystallographic orientation is important in MD simulations of CS, previous studies have shown that key phenomena like amorphization and Shockley partial dislocation behavior are unaffected by the chosen orientation for particle and substrate [11,27]. Therefore, this study uses the representative [100] direction for both Al-Al and Fe-Fe systems, as it is not a close-packed orientation in FCC or BCC structures. These modeling parameters, including the sample dimensions and time step, are further supported by their consistent application in previous MD studies of similar impact-induced phenomena [8,28,29], where simulation domains of comparable dimensions have been shown to sufficiently capture the underlying deformation and microstructural evolution mechanisms, at a reasonable computational cost. To accurately simulate CS conditions, the substrate was kept stationary during particle impact [26]. Therefore, as illustrated in Fig. 1c, a 4 nm thick layer with zero momentum was considered at its bottom region. Additionally, to keep the substrate at ambient temperature, a 6 nm thermostat layer was placed between the fixed and the computational layers, leaving 20 nm for the thickness of the computational layer directly exposed to the particle impact. It is important to note that material’s external oxide layers were intentionally excluded from the model to isolate intrinsic atomistic deformation and bonding mechanisms under ideal clean-metal conditions. However, this simplification may result in an overestimation of metallurgical bonding and interfacial mixing, as oxide layers present in actual CS processes impede direct metal–metal contact. Consequently, the presented results should be interpreted as representing an idealized, upper-bound bonding scenario, rather than a precise quantitative prediction of experimental behavior. This assumption has been extensively utilized in prior molecular dynamics (MD) research [8,28,29]

All simulations, including model construction and the impact procedure, were conducted using the open-source software LAMMPS, developed by Sandia National Laboratories [30]. To define the interatomic interactions in Al-Al and Fe-Fe systems, the EAM–FS (Embedded Atom Model–Finnis–Sinclair) potential function developed by Mendelev et al. [31] was used. The EAM–FS potential was employed for the Fe–Al system in the present study. This potential has been reported to reproduce the structural and thermodynamic properties of Fe–Al intermetallic compounds with reasonable accuracy [[32], [33], [34]]. Furthermore, it has been widely used in molecular dynamics studies of solid–solid and solid–liquid Fe–Al interfaces [[35], [36], [37]]. Since the formation of metallurgical bonding during cold spray is governed by interfacial processes such as atomic mixing and interfacial evolution, the EAM–FS potential was considered suitable for the objectives of the present work. In this simulation, atomic positions and velocities were updated at each timestep by solving the velocity-Verlet integration algorithm and computing the derivatives from the relevant functions [38]. The initial velocities of atoms were determined according to the Maxwell-Boltzmann distribution at specified temperatures for the substrate and the particle. The equations of motion were integrated using a time step of 1 fs to ensure numerical stability and accuracy. Since the atoms in the initial configuration are not in equilibrium or minimum-energy state, it is necessary to first bring them to equilibrium positions and velocities before impact simulation. Therefore, the energy equilibration protocol was defined as follows: initially, the system energy was minimized using the Conjugate Gradient (CG) algorithm. Subsequently, the system was equilibrated for 10 ps under fully Periodic Boundary Conditions (PBC) in all three directions using the NPT ensemble at zero pressure, while maintaining the substrate and the particle at room temperature. The temporary application of fully periodic boundaries during equilibration was intended solely to eliminate residual lattice stresses and obtain a fully relaxed crystalline structure prior to impact [39]. After this stage of the equilibration, the boundary condition along the z-direction was changed to non-periodic to create a free surface for particle impact, while periodicity was maintained in the lateral directions. Following this stage, a second equilibration step was carried out using separate NVT ensembles (constant number of atoms, volume, and temperature) to the particle and substrate, allowing their free surfaces to relax prior to impact. During this stage, the Al substrate was maintained at 300 K, while the Fe particle was heated to 673 K to approximate the carrier-gas temperature typically encountered in CS processes.

For the impact simulation, the particle impacted the substrate at a constant velocity of 1200 m/s. This velocity was selected to be higher than the critical velocity yet lower than the erosion velocity, ensuring particle bonding in both systems and enabling a consistent basis for comparison [40,41]. It is worth noting that the impact velocity used in this study was chosen within the range commonly reported in molecular dynamics simulations of CS particle impact [28,42,43]. Since the present work focuses on the microstructural evolution occurring at the particle-substrate interface under bonding conditions, the selected velocity was intended to fit within the deposition window and thus facilitate analysis of the underlying deformation and interfacial mechanisms. The impact velocity employed in the Al–Al and Fe–Fe systems gives rise to strain rates of approximately 6 × 10¹¹ s⁻¹ and 3 × 10¹¹ s⁻¹, respectively, both of which are consistent with the strain rate regimes reported in previous MD simulations of high-velocity impact processes [43]. It has been demonstrated that supersonic particle impacts on a substrate in MD simulations occur under the Adiabatic Shear Instability (ASI) [9,14]. More precisely, these conditions involve the application of extremely rapid and intense loading on the substrate surface, leading to large temperature and stress gradients as well as localized energy transfer. Under such circumstances, the rate of heat generation exceeds the rate of its dissipation, resulting in the retention of heat in the particle-substrate interface region and causing local softening of the material [44]. Therefore, during the impact simulation, the NVE ensemble (where the number of atoms, volume and energy of the system remain constant) was used for both the particle and the substrate, enabling a clear observation of the stress and temperature increase within the system.

Analysis tools in OVITO software [45] were employed at all stages of equilibration and impact simulations. Crystalline defects such as SFs, Twin Boundaries (TBs) and microstructural changes based on crystalline lattice differentiation were monitored at each stage using the Polyhedral Template Matching (PTM) [46] analysis tool. This analysis distinguished atoms belonging to different crystalline phases with unique colors. It performs more accurately than Common Neighbor Analysis (CNA) and adaptive CNA in processes with high thermal fluctuations, as it calculates local lattice orientation and elastic deformation gradients [46]. All dislocations in this study were identified using the Dislocation Extraction Algorithm (DXA) algorithm [47]. This analysis determines the Burgers vector and type of dislocations. The formation of new grains resulting from recrystallization was monitored using Grain Segmentation Modifier [48].

In MD approach, temperature is directly related to the kinetic energy of atoms and can be computed from atomic velocities along three spatial directions, atomic mass, and fundamental physical constants. However, the translational motion and overall displacement of the center of mass can lead to an artificial and unrealistic overestimation of the local temperature. To address this, following the approach presented by Daneshian et al. [49], the translational and rotational components of the center-of-mass motion were subtracted from the velocity calculations. This correction ensures that the large instantaneous atomic velocities arising from the impact event are not directly interpreted as thermal energy; rather, they are smoothed through a statistical averaging procedure to yield a physically meaningful approximation of the local temperature for each atom.

Following the methodology of Reddy et al. [14], the local temperature of each atom was determined using a statistical ensemble average of the thermal vibrations of neighboring atoms within a spherical region of 10 Å in diameter. It should be noted that the resulting local temperature values may exhibit a degree of sensitivity to the size of the selected averaging region. The spatial distribution and evolution of these thermal zones were subsequently analyzed and visualized using OVITO software.

## 3 Results and discussion

### 3.1 FCC system: phase transformations upon impact

**Fig. 2:**
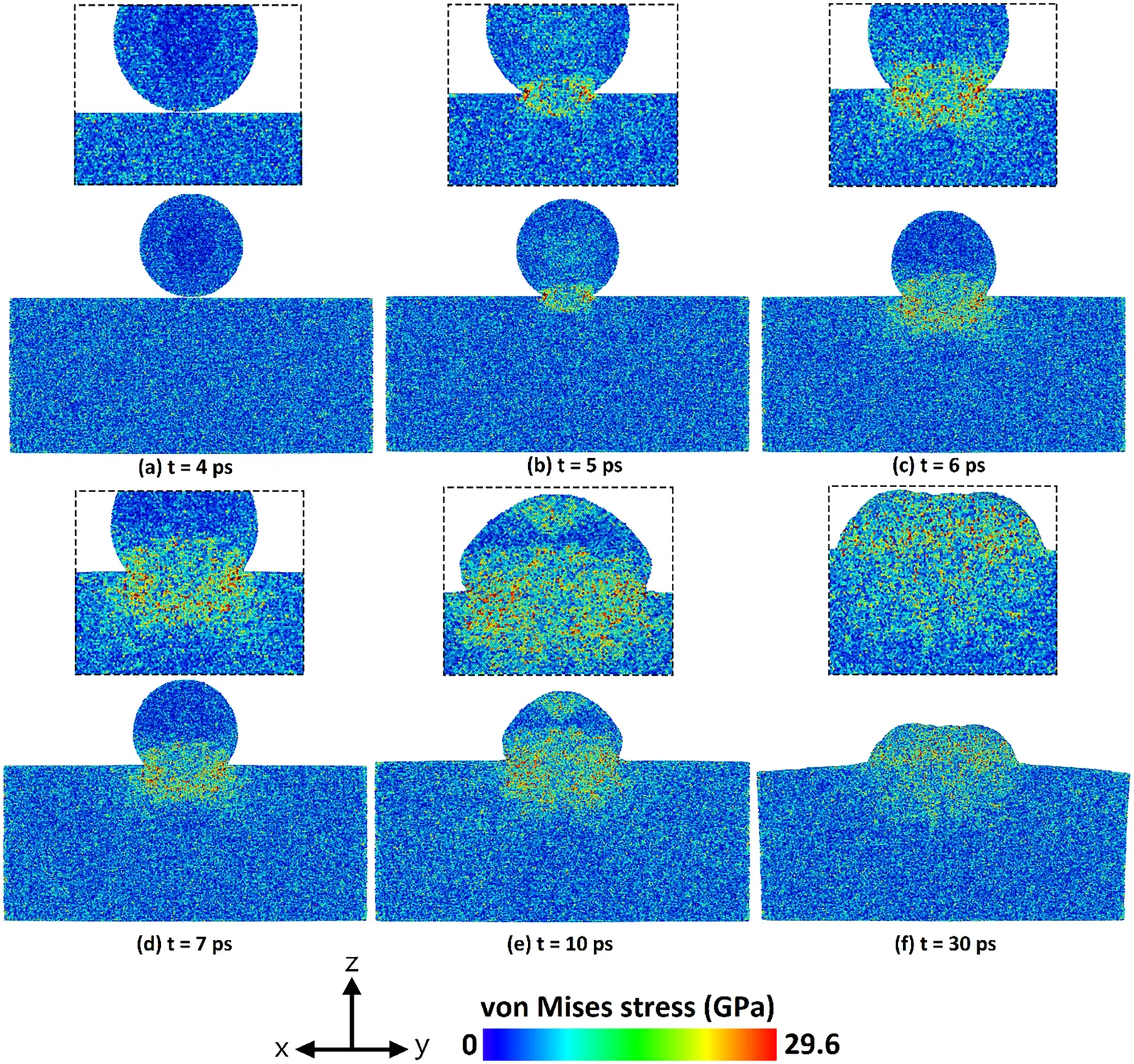
Evolution of von-Mises stress distribution maps of Al-Al system during impact: (a) 4 ps, (b) 5 ps, (c) 6 ps, (d) 7 ps, (e) 10 ps, and (f) 30 ps.

Understanding how impact energy is accommodated in FCC particle-substrate systems is essential for clarifying bonding mechanisms in CS. Accordingly, this section analyzes the evolution of stress, plastic deformation, and associated phase transformations in an Al-Al system during impact. As a first step, Fig. 2 illustrates the differentiation of atoms at various instants of impact, based on the distribution of von-Mises stress throughout the microstructure.

During the initial instants of impact, energy is concentrated at the interface between the particle and the substrate, with the von-Mises stress reaching a maximum of 29.6 GPa in this area. Due to the elevated stress concentration, the initial microstructural changes are expected to occur in this region (refer to Figs. 2a-c). Over time, the stressed regions expand further from the interface. As the particle continues to penetrate to the substrate (Figs. 2d-f), it absorbs more of the stress caused by the impact than the substrate, while in the substrate the impact energy spreads over a larger number of atoms.

**Fig. 3:**
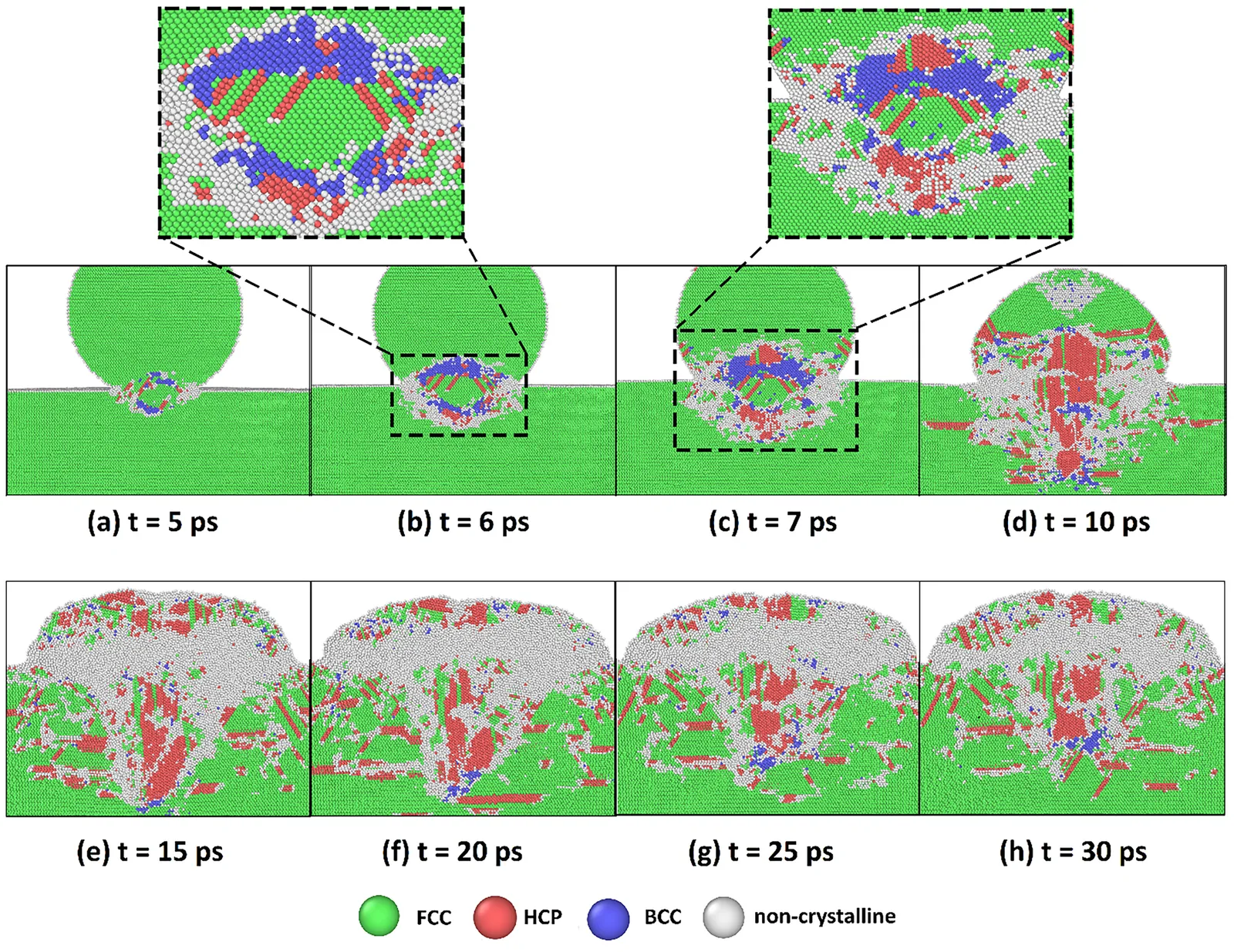
PTM analysis presenting the phase evolution of Al-Al system at various time intervals: (a) 5 ps, (b) 6 ps, (c) 7 ps, (d) 10 ps, (e) 15 ps, (f) 20 ps, (g) 25 ps, and (h) 30 ps.

Fig. 3 tracks how the microstructure of the Al-Al system evolves over different timesteps. As illustrated in Figs. 3a-c, the microstructure within the interface region, extending over a few atomic layers, can be classified into four states: (i) the liquid-like region, where atoms are distributed in a fully random and disordered arrangement. During the initial instants of impact, this microstructure is considered a collection of molten or liquid-like atoms. Unlike a crystalline structure, these atoms lack long-term ordering [50], thereby facilitating atomic diffusion between particle and the substrate. (ii) SF planes, which form mostly at 45° relative to the impact direction, gradually expand within the interface region. Such crystalline defects, represented as red atoms in Fig. 2, are uncommon in Al due to its high SFE (∼160 mJ/m^2^ [51]); however, under severe plastic deformation, the high energy impact drives the expansion of SF planes; and (iii) Solid-state phase transformation from FCC to BCC is another phenomenon observed at the interface during the early instants of impact.

**Fig. 4:**
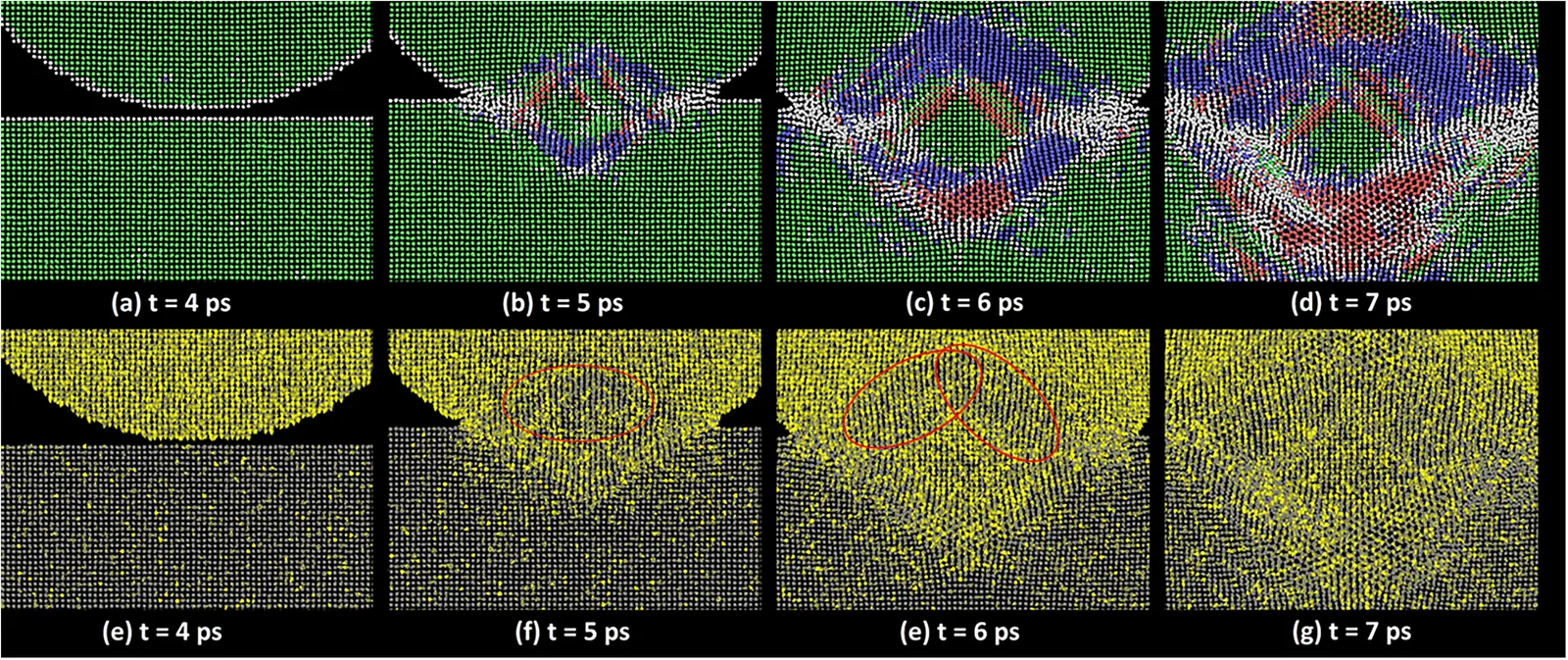
(a–d) Phase analysis performed using Common Neighbor Analysis (CNA). (e–g) Displacement trajectory lines showing the FCC-to-BCC phase transformation in the Al–Al system.

Al atoms are seldom found in a crystalline BCC structure. According to Abdolrahim et al. [21], two mechanisms can lead to this phase transformation in Al: first, the cross-interaction of slip (SF) planes, and second, a direct FCC to BCC lattice conversion. Such phase transformations are only feasible under extreme loading rates, and the resulting BCC phase is highly unstable, disappearing after load removal as Al atoms rearrange between the FCC and BCC structures. In our MD simulation, the stress and energy induced by the impact are sufficient to facilitate phase transformation at the particle-substrate interface. We propose that the observed FCC–BCC phase transformation may play a significant role in the bonding process. First, the transformation may contribute to the dissipation of impact energy. Second, as the BCC phase is generally less deformable than the FCC phase, it may reduce atomic mobility and hinder the penetration of particle atoms into the substrate. To shed more light on the observed phase transformation effect on the bonding process, the microstructural evolution of a 10 nm thick slab along the (110) view was analyzed at time steps ranging from 4 to 7 ps, the interval during which phase transformation reaches to its maximum extent. As shown in Figs. 4a–d, a layer of BCC crystal structure forms simultaneously in both particle and the substrate and progressively thickens over time. In Figs. 4e–h, displacement vectors indicating the direction of atomic motion relative to the previous time step are presented. The BCC phase layer exhibits significantly restricted atomic mobility, effectively acting as a barrier that impedes the downward motion of the FCC atoms located above it toward the substrate. It can therefore be concluded that the transiently formed BCC structure temporarily disrupts the energy transfer pathway from the particle to the substrate, negatively affecting bonding. At 10 ps (Fig. 3d), the BCC phase transforms into an HCP structure. During this period, a significant portion of the particle undergoes crystalline restructuring, while SF planes extend into the deeper regions of the substrate. This observation clearly demonstrates the co-deformation phenomenon, which has been reported previously in the CS modeling of similar particle-substrate systems [52].

**Fig. 5:**
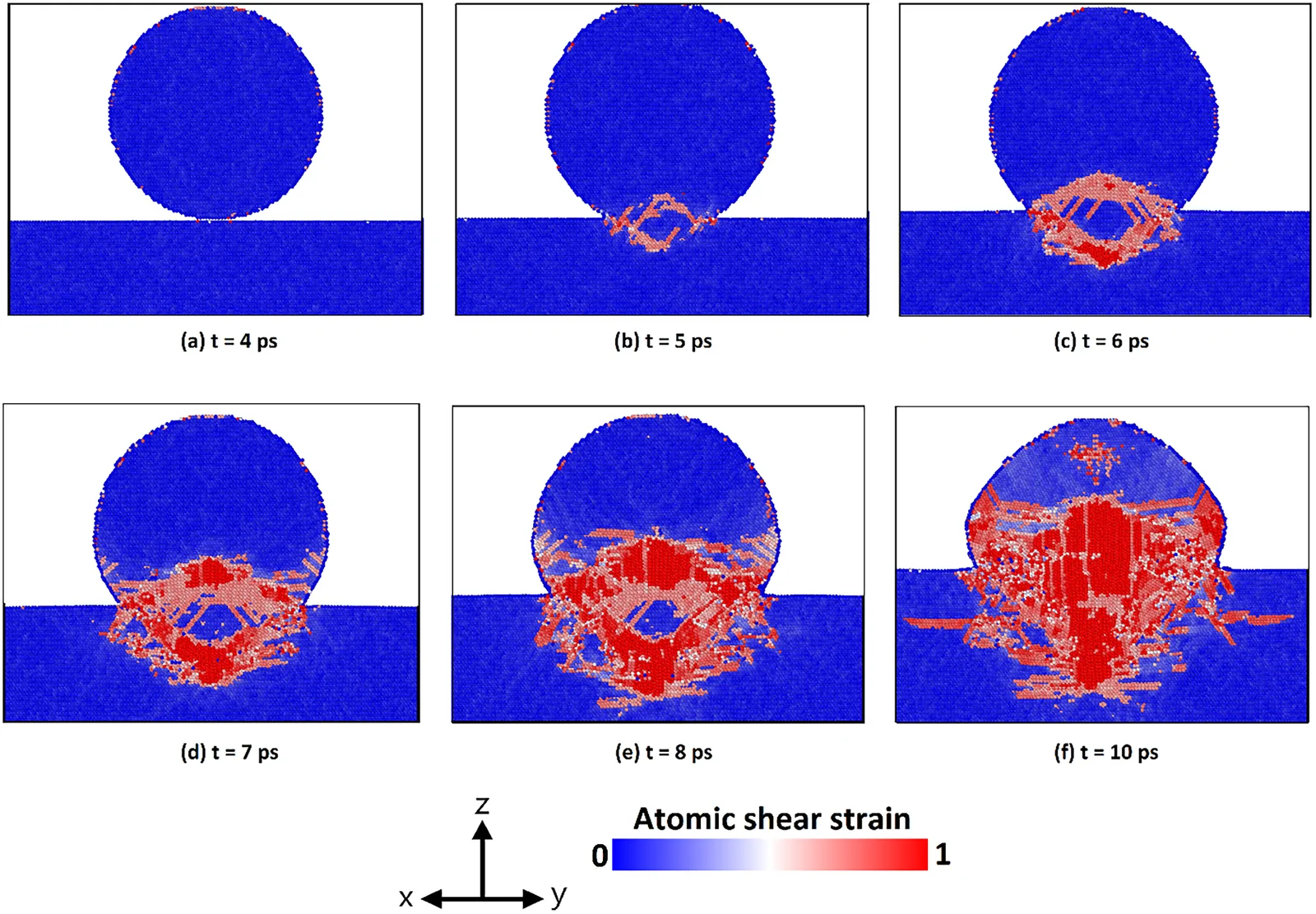
Evolution of atomic shear strain distribution for Al-Al system at different time intervals: (a) 4 ps, (b) 5 ps, (c) 6 ps, (d) 7 ps, (e) 8 ps, and (f) 10 ps.

From 15 to 30 ps, particle penetration into the substrate stops, yet plastic deformation persists through nucleation, movement, and expansion of SF planes (see Figs. 3e-h). The stress distribution map presented in Fig. 2e reveals a notable stress concentration at the top of the particle, which can be interpreted in light of the microstructural evolution shown in Fig. 3d. At 7 ps, corresponding to the early stages of impact (Fig. 2d), the energy imparted by the collision remains considerably high, and the crystalline disorder along with the molten zones generated by the impact facilitate particle penetration into the substrate, such that the particle encounters relatively low resistance during this stage. At this point, the morphology of the upper portion of the particle remains essentially spherical. However, during the intermediate and later stages of impact, the particle can no longer penetrate the substrate as readily as in the initial stages, owing to the increasing deformation resistance of the substrate, the progressive dissipation of kinetic energy, and the transmission of the impact-induced stress wave toward the upper portion of the particle. This stress wave induces deformation in the particle itself, causing it to deviate more from its original spherical morphology, as evidenced by the final microstructure shown in Fig. 2f. Accordingly, the microstructure presented in Fig. 2e represents a transitional state marking the shift from the early to the intermediate stage of impact, during which stress concentration arises from the onset of deformation-induced crystalline disturbance at the top of the particle, as further confirmed by the dislocation distribution shown in Fig. 3d. Recrystallization within both the particle and the substrate is distinctly visible during the final stages of the impact. In regions further from the interface, dislocation arrangements and their overlapping result in the formation of subgrains during the recovery process, manifesting distinct grains within the single-crystal microstructure of the particle and the substrate (indicated by black arrows). Reddy et al. [53] referred to these newly formed grains, characterized by an abundance of SF planes, as "sheared grains", attributing the emergence of SF planes within these grains to the high shear strain present. Zhao et al. [54] suggested that plastic deformation under extreme strain rates in metals occurs through solid-state phase transformations, which limits the material's capacity to accommodate deformation via dislocations, twinning, and other crystalline defects. Our MD simulations, on the other hand, indicate that the elevated plastic strain rate at the onset of impact, particularly at the interface, triggers atomic interaction and coalescence mechanisms that lead to both solid-liquid transformations, such as localized partial melting, and solid-solid phase changes, including FCC-to-BCC and FCC-to-HCP transitions. The primary catalyst for these solid-state phase changes is likely the intense shear strain applied to the crystalline structure of Al. Figs. 5a-f illustrate the shear strain distribution map prior to impact, as well as during the initial and mid-stages up to 10 ps. As depicted in these figures, shear bands are prominently formed in the regions where crystalline phase transformations occur. Over time, the areas of accumulated shear strain increase, reaching their peak at 10 ps. These findings align with the microstructural changes presented in Fig. 3.

**Fig. 6:**
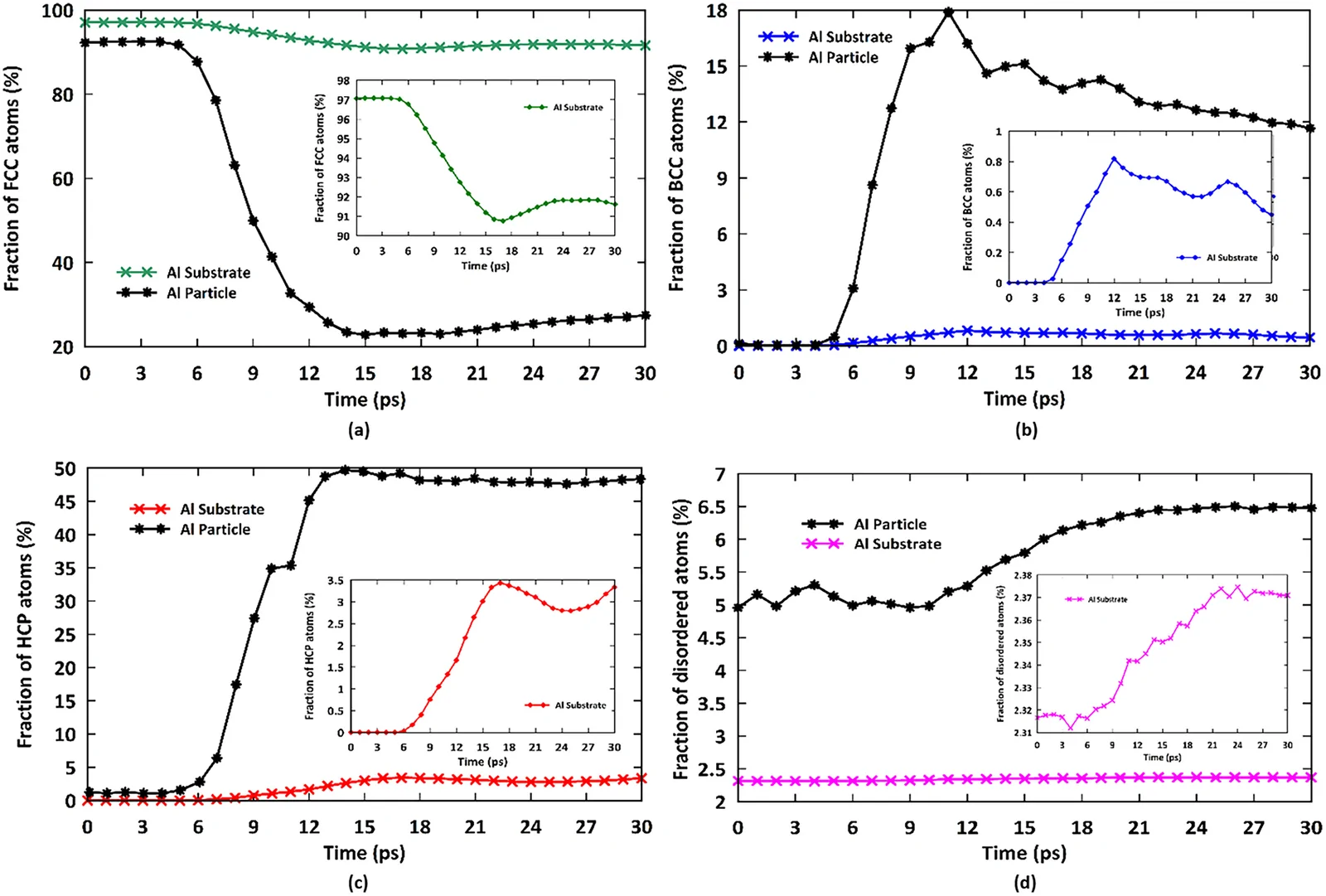
Quantitative analysis of microstructural changes in the Al-Al system over time using PTM analysis: fraction of (a) FCC, (b) BCC, (c) HCP, and (d) disordered atoms.

Fig. 6 illustrates the quantitative progression of phase transformations for the Al-Al system. Figs. 6a–6d show more pronounced phase transformations in the particle rather than the substrate, in line with the greater stress concentrations in the particle during impact (see Fig. 2). As shown in Fig. 6a, upon impact, between 6 and 10 ps, the fraction of FCC atoms within the particle decreases sharply by approximately 70% over 4 ps. Such significant crystal structure changes are evident in intense plastic deformation resulting from shock-based loading regimes. Fig. 6b indicates that during this period, the proportion of BCC atoms reaches around 17%. Notably, the prevalence of this crystalline phase fades over time. In the final microstructure, a portion of the BCC phase persists, but its atomic distribution no longer forms clusters and therefore cannot be classified as a continuous phase. In contrast, extensive regions of SFs develop throughout different areas of the particle, serving as a substantial replacement for the original FCC phase. These SF planes remain remarkably stable in the final microstructure, constituting about 50% of the particle's atoms.

As previously mentioned, the presence of such a high fraction of SFs in the Al structure is unexpected due to its high SFE. Since the mechanism of HCP formation involves the reordering of atomic layers through partial dislocation slip, a logical correlation is anticipated between this type of dislocation and the high fraction of HCP atoms. This correlation is further elaborated in the subsequent section. According to Fig. 6d, changes in disordered atoms, which represent melted and pre-melted atoms, evidence that at the earliest instances after impact, these atoms are limited in number and confined to only a few atomic layers at the interface. Thus, it can be concluded that in the Al-Al system, solid-solid phase transformations play a more significant role in the microstructural changes of both particle and substrate compared to solid-liquid transformations. Over time, the fraction of disordered atoms increases by approximately 1.5%, which can be attributed to the formation of GBs during the recrystallization process.

### 3.2 FCC system: role of dislocation activity on bonding strength

**Fig. 7:**
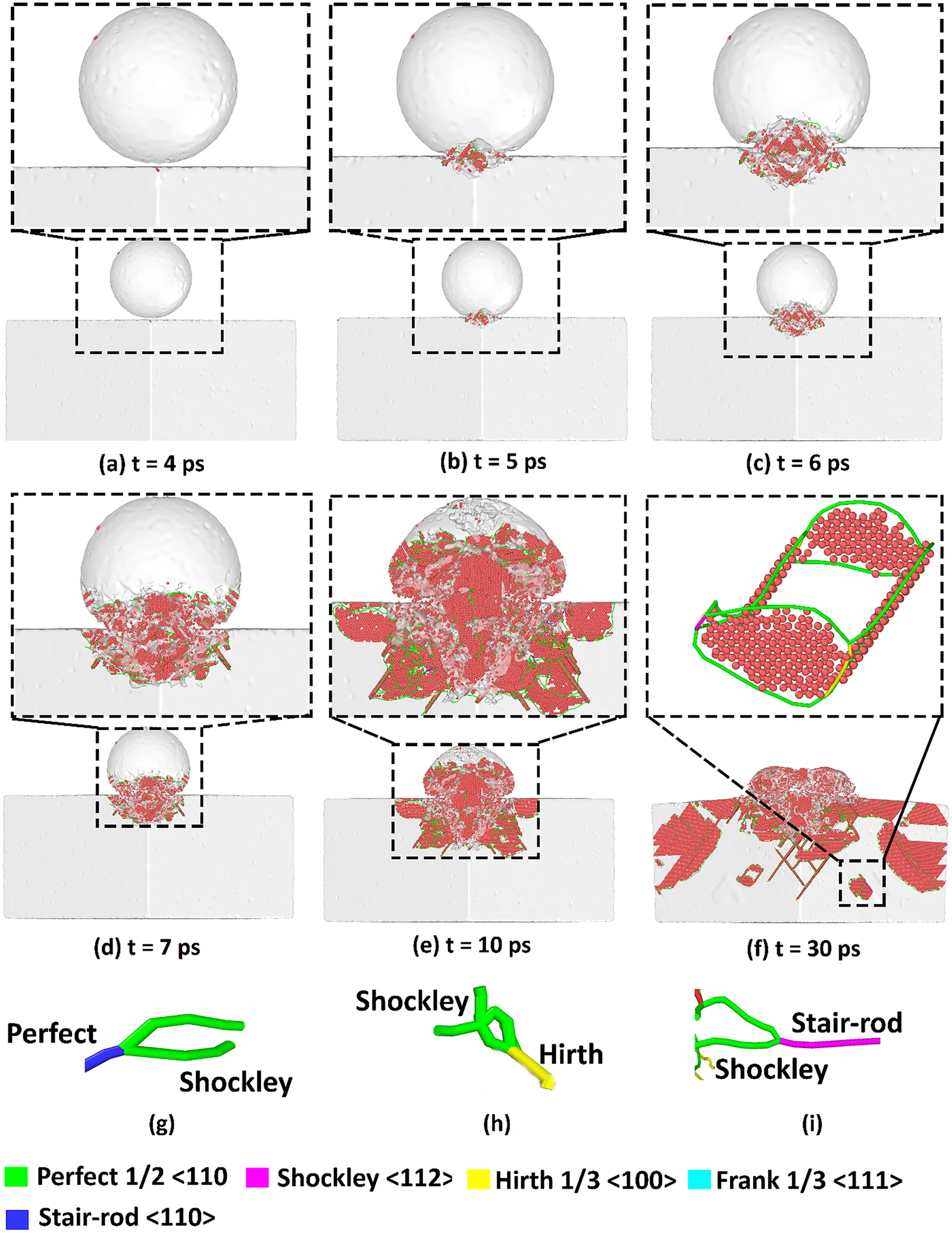
DXA analysis in the Al-Al system at different time intervals: (a) 4 ps, (b) 5 ps, (c) 6 ps, (d) 7 ps, (e) 10 ps, and (f) 30 ps. (g-i) Partial dislocation interactions at the interface in the final microstructure.

Plastic deformation in crystalline metals cannot occur without dislocation activation [55]. In regimes of slow to moderate plastic deformation, nucleation, slip, and pile-up of dislocations significantly influence the material’s mechanical properties and strength [56]. Specifically, during severe plastic deformation at high strain rates, a substantial number of dislocations is generated in an extremely short period, and their entanglement complicates the deformation process. To investigate the role of dislocations in microstructural changes and bonding strength in an Al-Al system, the interface microstructure was monitored at various stages of impact using DXA analysis (see Figs. 7a to 7e).

It is observed that SF planes develop extensively within the FCC structure, and there is a substantial nucleation of Shockley partial dislocations (indicated by green lines and 1/6 < 112> Burgers vectors) at the particle-substrate interface. As presented in magnified inset in Fig. 7f, these partial dislocations are unable to facilitate perfect slip within the crystalline FCC lattice; therefore, they modify the stacking of atomic planes to form HCP faults [57]. Consequently, there is a direct correlation between the large number of SFs and the elevated density of Shockley partial dislocations. Previous studies have shown that under high-rate deformation, perfect dislocations in the FCC lattice decompose into Shockley partial dislocations in accordance with Frank’s energy criterion [58]. For instance, in the interface region of the current sample (see Fig. 7g), the decomposition reaction of a perfect dislocation can be expressed as:

(1) $$\frac{1}{2}\langle 0\bar{1}\bar{1}\rangle \to \frac{1}{6}\langle \bar{1}\bar{2}\bar{1}\rangle +\frac{1}{6}\langle 1\bar{1}\bar{2}\rangle$$

In addition to the direct nucleation of Shockley dislocations, the decomposition of perfect dislocations also contributes to their increased density, thereby facilitating FCC-to-HCP phase transformation. SF planes, by changing the slip systems, act as obstacles to dislocation slip path, resulting in strain hardening [[59], [60], [61]]. Thus, a high density of such crystallographic defects at the interface can enhance bonding strength. Previous studies have indicated that a significant density of Shockley partial dislocations occurs during CS deposition in FCC metals with medium and low SFE, such as Cu, Ag, and Ni [62]. However, the present study demonstrates that Al, despite its high SFE, can also exhibit analogous dislocation decomposition behavior.

The effects of Shockley dislocation entanglement extend beyond forest hardening. Their interactions, occurring over extremely short times, lead to the formation of immobile dislocations, which, due to their Burgers vectors, cannot glide and are therefore considered locked. For example, DXA analysis at 30 ps shows that, at the interface region, two Shockley partial dislocations combine to form a Hirth dislocation (Fig. 7h), based on the following reaction:

(2) $$\frac{1}{6}\langle \bar{1}21\rangle +\frac{1}{6}\langle \bar{1}\bar{2}\bar{1}\rangle \to \frac{1}{3}\langle \bar{1}00\rangle$$

Simultaneously, the merging of two additional Shockley partial dislocations can result in the formation of a stair-rod dislocation with a partial Burgers vector (Fig. 7i), as the following reaction:

(3) $$\frac{1}{6}\langle \bar{1}21\rangle +\frac{1}{6}\langle 21\bar{1}\rangle \to \frac{1}{6}\langle 1\bar{1}0\rangle$$

These dislocations possess slip directions that differ from the packed slip directions of the FCC lattice, remaining stationary within the interface and serving as obstacles to other dislocations [63,64]. Consequently, they can enhance the interface strength in this CS system. Fig. 7F demonstrates that, after impact, substantial concentrations of SFs and dislocations are present both at the interface and areas far from it. This shows that stress waves from the impact in FCC Al cause significant microstructural changes, extending well beyond the immediate contact region.

**Fig. 8:**
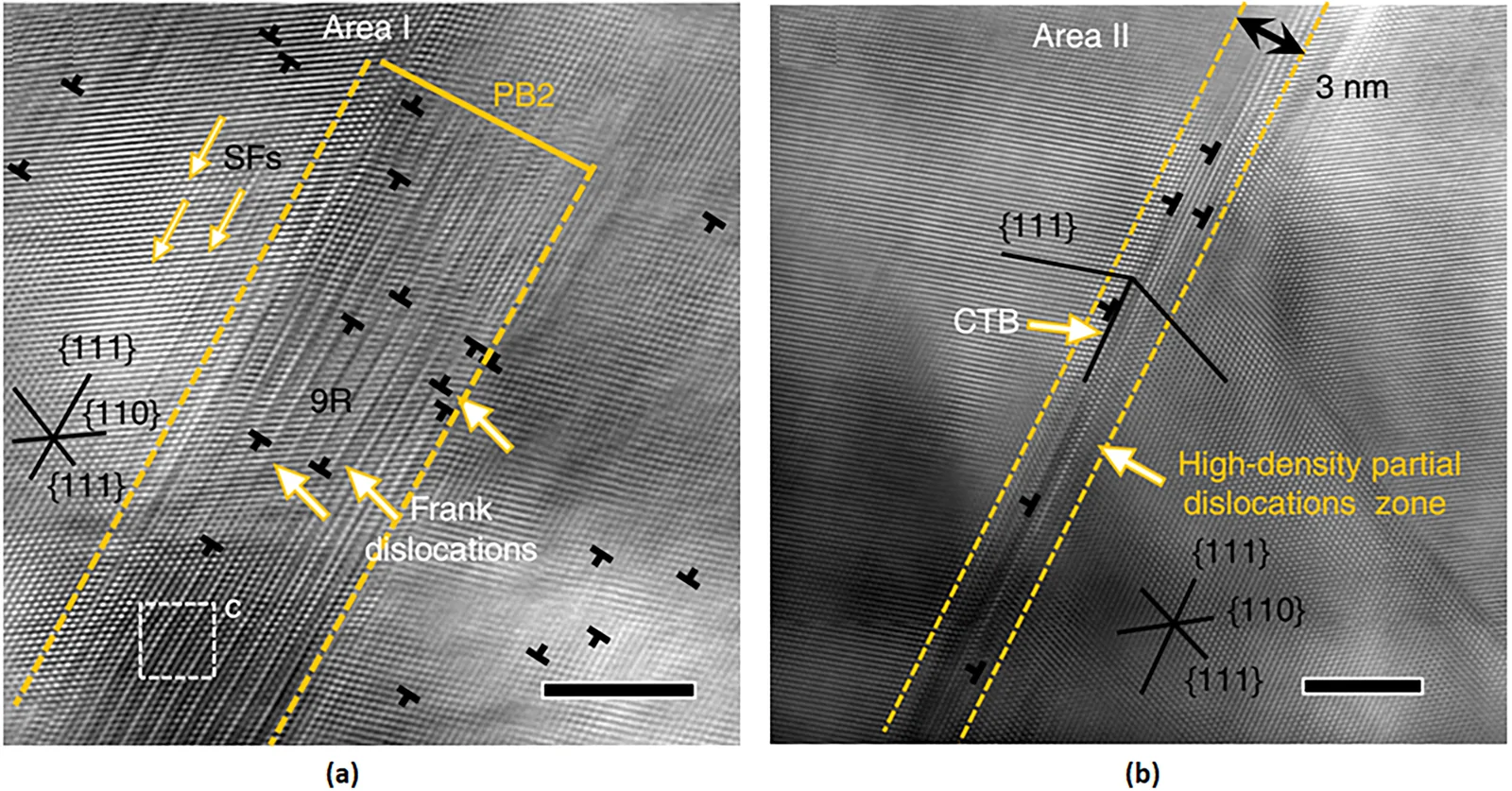
The HRTEM micrograph of projectile impact induced 9R phase in UFG Al films: (a) 9R phase, SFs, and Frank dislocation detection, and (b) High density of partial dislocations [65]. All data have been reproduced with permission.

Plastic deformation in Al is typically dominated by dislocation activity. However, the extremely high strain rates generated during high velocity impact can promote deformation also by twinning. In this context, Xue et al. [65] employed the Laser-Induced Projectile Impact Test (LIPIT) technique to investigate the severe plastic deformation behavior of ultrafine-grained (UFG) Al thin films under supersonic impact of monodispersed silica microspheres (∼3.7 µm in diameter) launched at the high velocity of approximately 600 m/s .Fig. 8 presents the microstructural features of the thin film obtained via High-Resolution Transmission Electron Microscopy (HRTEM). Figs. 8a and 8b present a typical deformation twin, with orange dashed lines indicating 9R phase and two Coherent Twin Boundaries (CTBs). Multiple SFs are observed in Fig. 8a on the left side of the large 9R region. In addition, numerous Frank partial dislocations were identified within the 9R phase. As indicated in Fig. 8b, a deformation-induced CTB contains a high density of Shockley partial dislocations. A comparison with current simulation results shows that the Shockley partial dislocations, Frank dislocations, and SFs observed experimentally during CS align with predictions from other high-velocity impact processes.

**Fig. 9:**
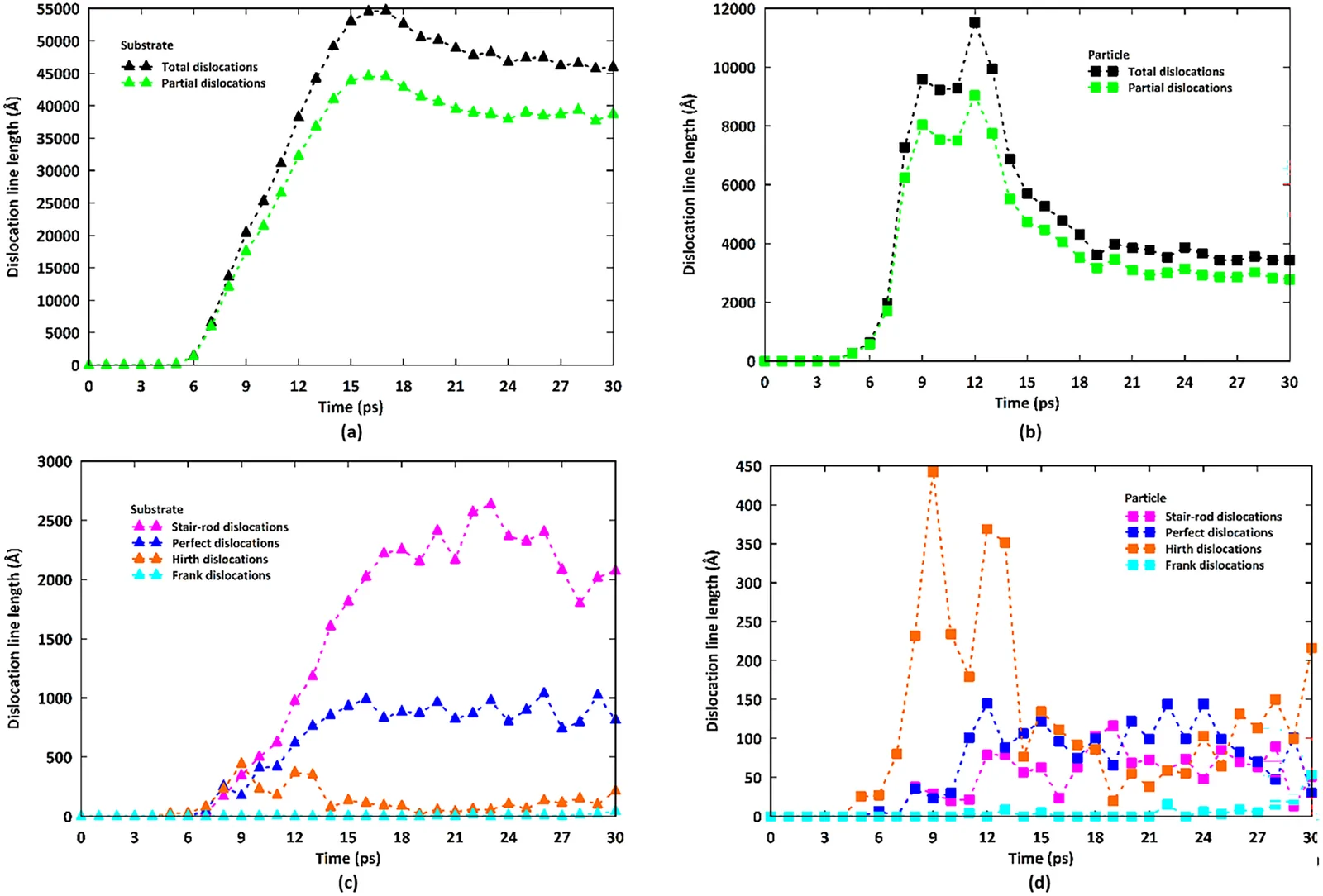
Quantitative DXA analysis in the Al-Al system over time: (a) total and partial dislocations in the substrate, (b) total and partial dislocations in the particle, (c) partial and lock dislocations in the substrate, and (d) partial and lock dislocations in the particle.

Fig. 9 provides quantitative comparisons of dislocation line lengths at varying stages of impact. Figs. 9a and 9b demonstrate that Shockley partial dislocations constitute a significant portion of the total dislocations in both substrate and the particle. Dislocation density experiences a sudden increase during the initial moments of impact (approximately 6–13 ps) and continues to rise during the intermediate phase (14–18 ps) as the particle penetrates the substrate, ultimately decreasing in the final stage due to dislocation slip and recrystallization. Changes in locked dislocations closely correlate with interactions among partial Shockley dislocations, rendering their behavior less predictable. Nevertheless, stair-rod and Hirth dislocations are the available locked dislocations in the substrate and particle (see Figs. 9c and 9d). The low density of perfect dislocations can be attributed to their extensive decomposition into Shockley partial dislocations. The particle exhibits greater fluctuations in dislocation density over time compared to the substrate due to the more intense deformation, as illustrated in Fig. 7.

Tang et al. [8] have demonstrated that particle impact on a substrate generates a high density of dislocations near the interface, leading to pronounced strain hardening due to dislocation entanglement. In the context of the previously discussed interfacial deformation mechanisms, this dislocation accumulation enhances both the strength and toughness of the substrate locally. As a result, the formation of a dislocation “forest” in the interface region not only increases the resistance of the substrate to further deformation but also improves particle retention by mechanically anchoring the particle within a hardened interfacial zone, while preserving the overall mechanical integrity of the substrate. Therefore, it can be concluded that, in addition to the phase transformations outlined in Section 3.1, the density, characteristics, and interactions of dislocations at the interface are crucial to enhancing interfacial bonding. Specifically, dislocation locks formed by the interaction of Shockley partial dislocations can create a dense network of entangled dislocations in both substrate and the particle within the interface region, thereby significantly improving interfacial mechanical integrity.

Although the substrate and particle share identical material composition, an asymmetry emerges in the density and type distribution of dislocations between the two bodies. In the Al-Al system, Shockley partial dislocations account for the majority of dislocation content, as reflected in both the qualitative and quantitative results of Fig. 7, Fig. 9. Nonetheless, lock-type dislocations, namely Frank, Hirth, and stair-rod types arising from mutual interactions among Shockley partial dislocations, accumulate at notably higher densities in the substrate compared to the particle. Two factors contribute to this behavior: first, the larger volume of the substrate accommodates a substantially greater population of Shockley partial dislocations, raising the likelihood of mutual encounters among them; second, the wider slip space and superior dislocation mobility present in the substrate amplify the frequency of dislocation collisions, enabling secondary dislocation types to emerge from Shockley partial dislocation reactions.

### 3.3 BCC system: phase transformations upon impact

**Fig. 10:**
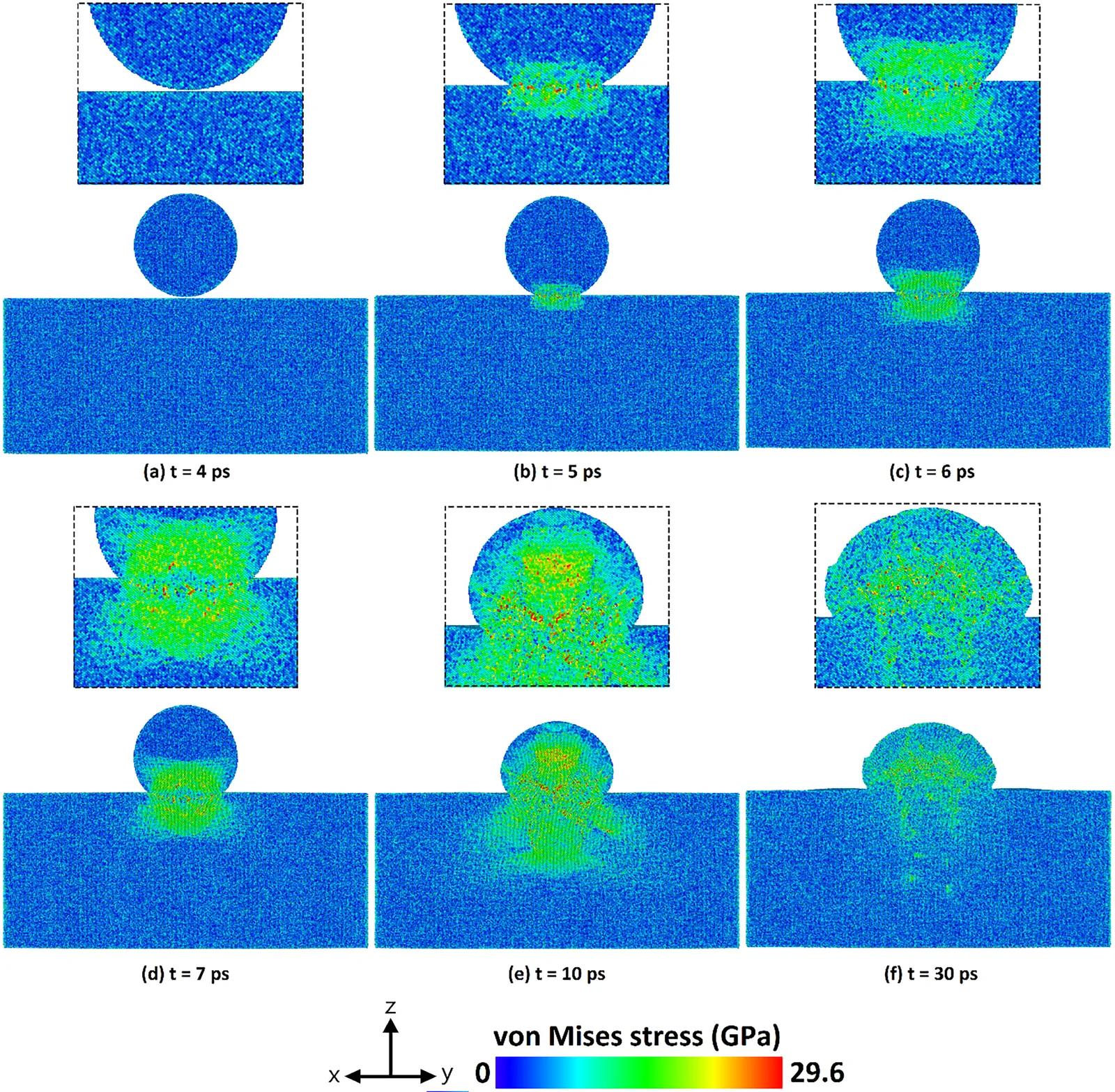
Evolution of von-Mises stress distribution maps of Fe-Fe system during impact: (a) 4 ps, (b) 5 ps, (c) 6 ps, (d) 7 ps, (e) 10 ps, and (f) 30 ps.

Similar to what observed for the FCC (Al-Al) system, the BCC (Fe-Fe) system also shows pronounced interfacial stress concentration early after impact (Figs. 10a-c). Over time, and during the intermediate and later stages of impact, larger portions of both particle and the substrate experience stress waves propagating from the interface region (Figs. 10d–f).

A comparison of the stress distributions at corresponding time points in Fig. 2, Fig. 10 reveals a fundamental distinction between Fe and Al. In BCC-structured Fe, stress is distributed across a larger number of atoms within both particle and the substrate; however, the fraction of atoms experiencing peak stress remains significantly smaller than in Al system. In contrast, FCC-structured Al exhibits a more spatially confined stress distribution, yet with considerably higher local stress magnitudes and intensities. This behavior can be attributed to the intrinsic differences in crystal structure between the two materials, which govern the mechanisms of stress partitioning and redistribution during high strain rate impact.

**Fig. 11:**
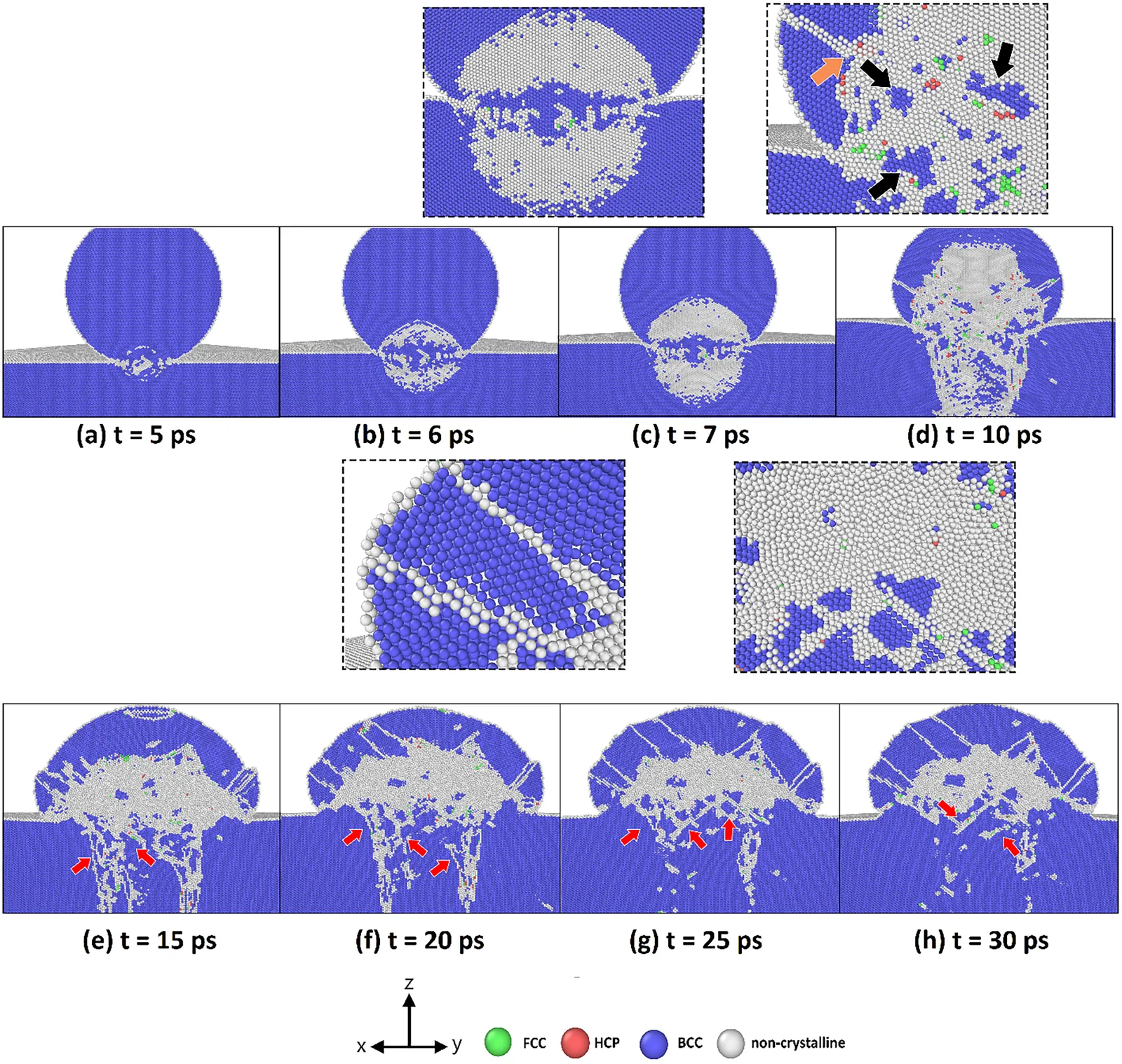
PTM analysis presenting the phase evolution results in the Fe-Fe system at various time intervals: (a) 5 ps, (b) 6 ps, (c) 7 ps, (d) 10 ps, (e) 15 ps, (f) 20 ps, (g) 25 ps, and (h) 30 ps.

Fig. 11 illustrates microstructural changes within the BCC system across different time steps using PTM analysis. During the initial stages of the impact (Figs. 11a-c), a considerable fraction of disordered atoms, which gradually expands over time, is visible at the interface. In Fig. 11d, dispersed HCP and FCC atoms can be observed; however, they cannot be regarded as either well-defined clusters or a continuous phase, and should instead be considered GB atoms. At 10 ps, nucleated grains within the disordered interfacial region are shown by black arrows, while an orange arrow refers to a twin formed in the particle. In BCC structures, due to the limited number of slip systems, plastic deformation proceeds predominantly via twinning rather than dislocation slip [66], compared to the Al-Al system. Nevertheless, dislocation activity plays a substantial role in microstructural evolution, as described in detail in the following section.

Stress resulting from the impact must be dissipated through plastic deformation to promote atomic bonding between the particle and the substrate, thereby enhancing interfacial strength. Consequently, the initially disordered atom-rich region at the interface transforms into newly formed grains at the center of the interface, leading to the formation of twins extending into farther regions (see Figs. 11e–h). According to previous studies [67,68], twinned regions can locally change crystal orientation, redirect dislocation slip paths, and thus increase material’s strength. Therefore, twins are interpreted as having a dual effect: on one hand, they facilitate plastic deformation, and on the other hand, they act as obstacles that can locally restrict plastic flow. Thus, the presence of twins near the interface (indicated by red arrows) can increase the strength of this region.

**Fig. 12:**
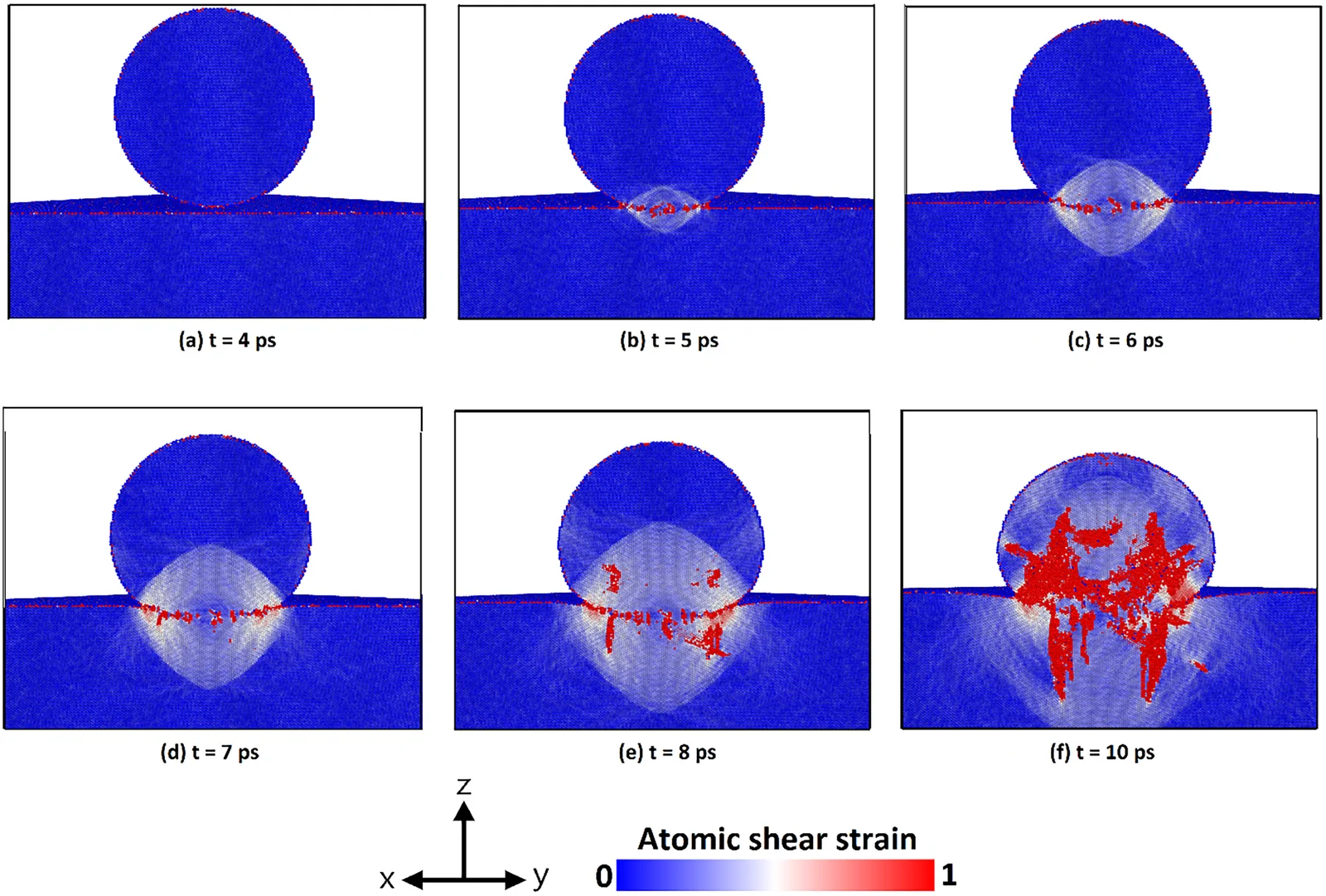
Atomic shear strain distribution map in the Fe-Fe system at different time steps: (a) 4 ps, (b) 5 ps, (c) 6 ps, (d) 7 ps, (e) 8 ps, and (f) 10 ps.

In general, compared to the FCC system, the BCC system’s impact energy is less effective at causing substantial plastic deformation or triggering phase transformations. This capacity is supported by the shear strain maps shown in Fig. 12, which reveal that strain is highly distributed within the particle and the substrate, leading to less localized concentration in the interfacial region. Furthermore, at 10 ps, when the stress reaches its maximum in both particle and the substrate, the regions with maximum shear strain are considerably smaller than those in the corresponding situation in the FCC system (see Fig. 5f).

**Fig. 13:**
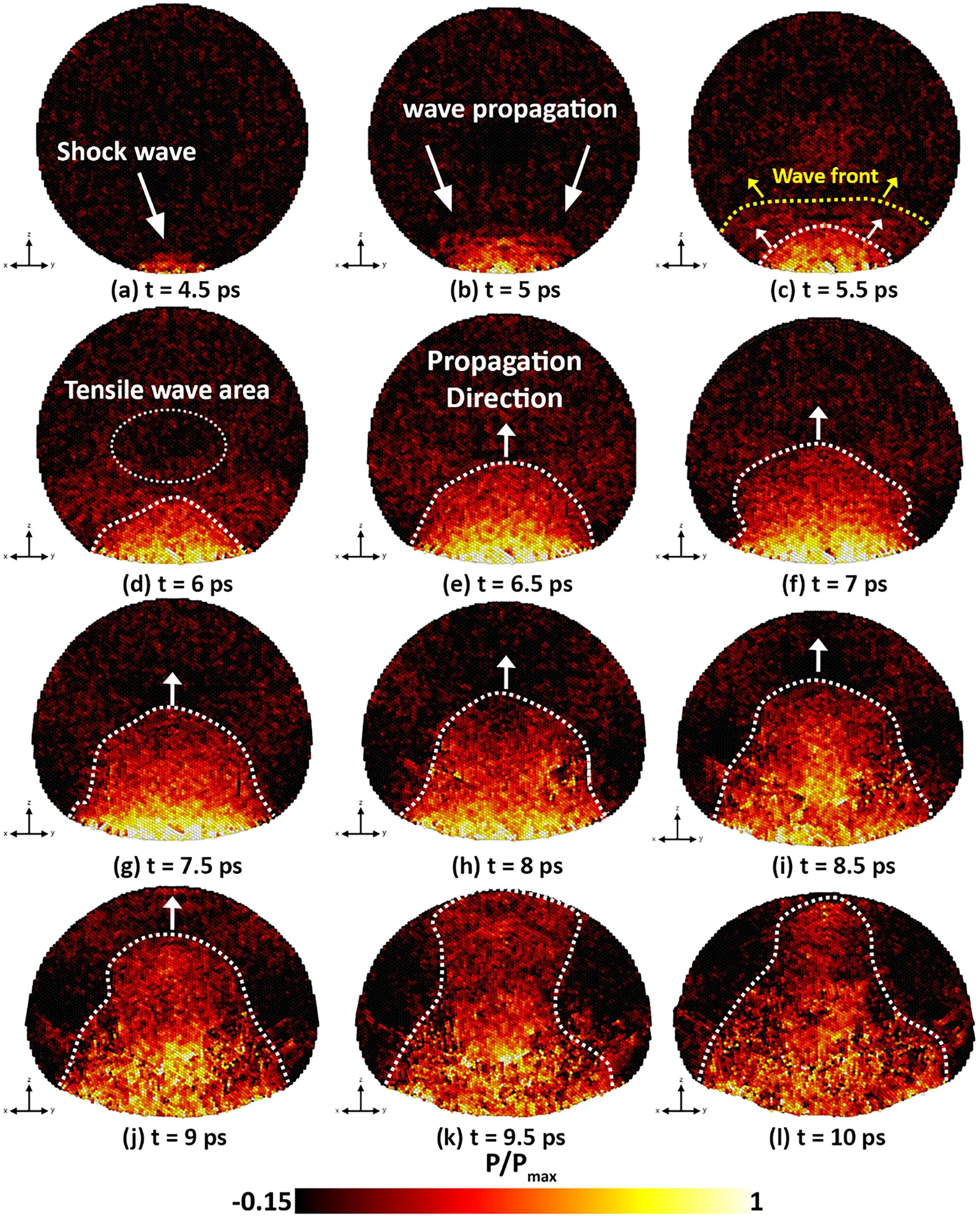
Temporal evolution of the normalized pressure field (P/Pmax) during Fe-Fe impact from 4.5 to 10 ps. Snapshots capture the generation of the initial shock wave at the impact interface, its propagation through the particle, and the subsequent formation of a tensile-wave region due to wave reflection and release processes. Dashed contours mark the advancing pressure-wave front. The progressive upward movement of the wave front demonstrates the redistribution of impact-induced stresses and the development of localized deformation, which contribute to the microstructural changes occurring during particle impact.

As observed in Fig. 12, the impact-induced stress waves are clearly reflected in the atomic shear strain distribution as well. To examine the evolution of these waves in greater detail, their propagation patterns within the particle are analyzed in the following section. Fig. 13 illustrates the normalized pressure distribution (P/Pmax) and wave propagation behavior inside the particle during impact with the substrate over the time interval of 4.5 to 10 ps. At the initial stage of impact, at t = 4.5 ps, a highly compressed region forms at the particle–substrate interface, indicating the onset of shock wave generation. At this stage, the shock wave remains localized near the contact region and does not yet propagate significantly into the particle interior. As time progresses to t = 5 ps, the shock wave begins to propagate upward inside the particle, while the maximum compressive pressure remains concentrated near the impact center. At t = 5.5 ps, a distinct wave front becomes clearly visible, exhibiting a semi-spherical propagation pattern governed by the particle geometry.

At approximately t = 6 ps, a lower-pressure region appears above the propagating wave front, corresponding to the formation and propagation of tensile waves generated by stress release and wave reflection phenomena. This observation indicates that, in addition to the intense compressive stresses, part of the particle experiences tensile stress conditions. Between t = 6.5 and 7.5 ps, the wave front continues propagating toward the upper region of the particle while the compressive zone expands significantly. During this period, the lower part of the particle remains under severe compression, suggesting a substantial increase in deformation rate and the activation of plastic deformation mechanisms.

At later stages, particularly between t = 8 and 9 ps, the initially smooth pressure distribution becomes increasingly non-uniform, and localized pressure fluctuations develop within the particle. These features are attributed to the interaction between compressive and tensile waves, multiple wave reflections, and local stress concentration effects. Finally, at t = 9.5 and 10 ps, the wave front reaches the upper region of the particle, while severe deformation and lateral spreading become evident near the particle bottom. The highly disturbed pressure field observed at this stage indicates intense plastic flow and extensive material deformation. The results demonstrate that the intensity and propagation dynamics of the shock wave play a dominant role in the deformation behavior of the particle, whereas the maximum localized pressure alone is not sufficient to explain the extent of plastic deformation.

**Fig. 14:**
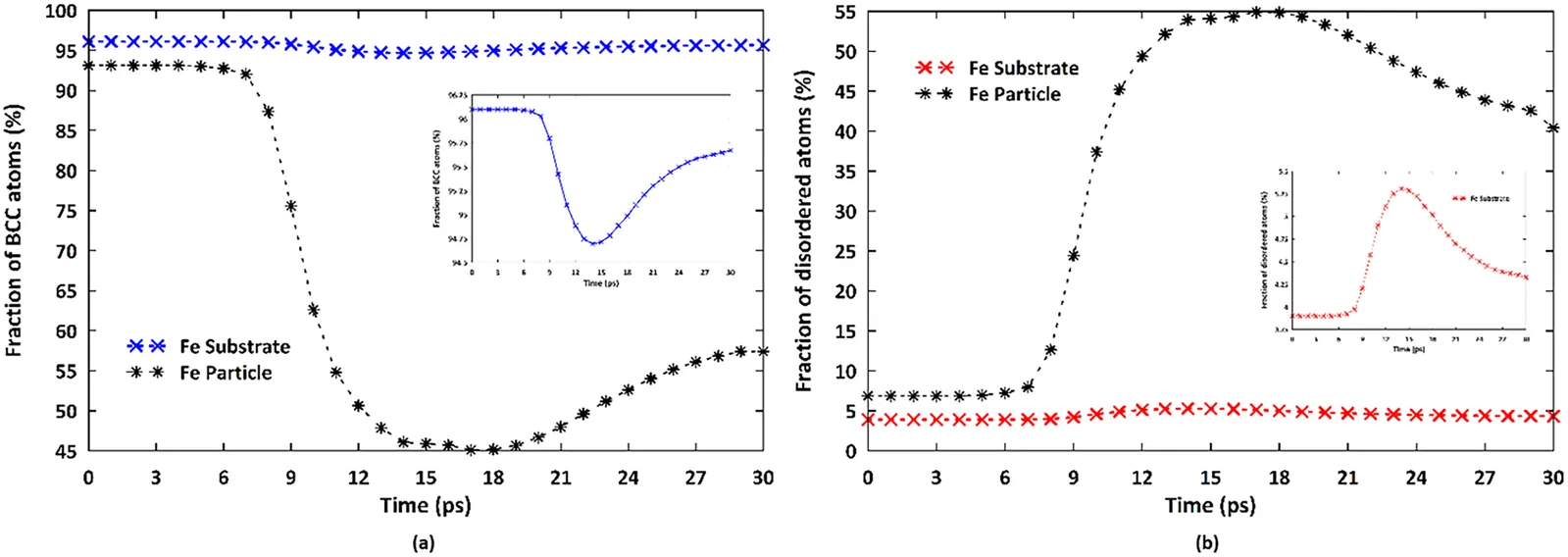
Quantitative analysis of microstructural changes in the Fe-Fe system over time using PTM analysis: volume fraction of (a) BCC and (b) disordered atoms.

Fig. 14 presents the quantitative evaluation of atoms undergoing phase transformation at various time steps during the impact. These findings suggest that, as observed in the FCC system, the substrate experiences only slight microstructural changes, while the particle displays the most significant structural variations.

During the initial instances of impact, nearly half of the atoms in the particle depart from the perfect BCC structure, forming disordered atoms. In the early impact stages, these atoms may appear as melted or pre-melted, but as time progresses into the intermediate and later stages, they manifest as disordered atoms along GBs and TBs due to cooling. Furthermore, comparison of Figs. 14a and 14b in the 20–30 ps indicate that some of these disordered atoms return to the BCC structure as new grains form. This phenomenon is reflected by the rise in the fraction of BCC atoms and decline in the fraction of disordered atoms within the particle over time. It should be noted that accurately distinguishing between melted, pre-melted, amorphous, and GB atoms over the short timescale of impact remains challenging. Nevertheless, the present findings suggest that attaining melting or pre-melting temperatures at the initial stage of impact can significantly enhance metallurgical bonding, in agreement with recent experimental studies [69,70].

### 3.4 BCC system: mechanisms of twin nucleation and thickening

**Fig. 15:**
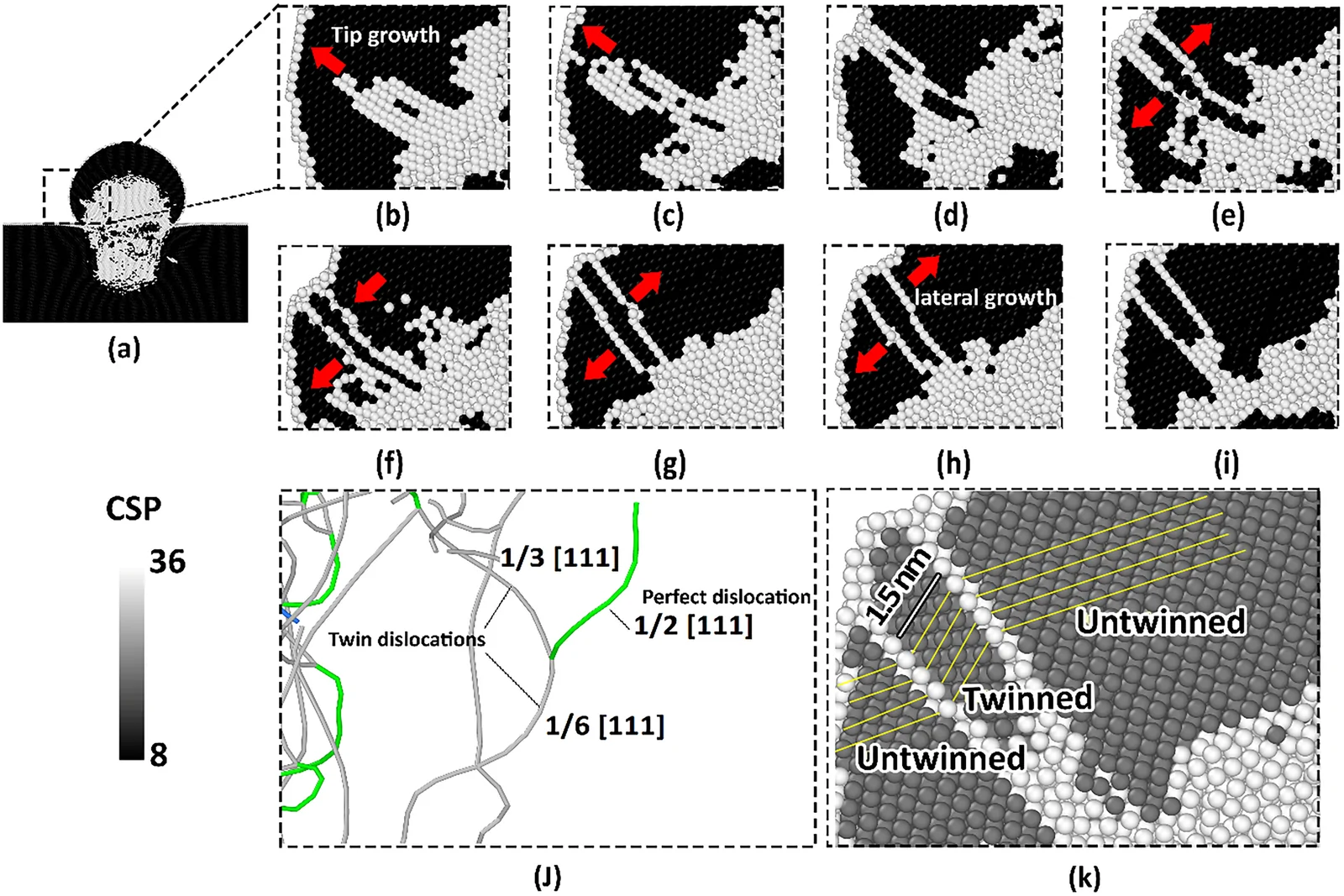
(a–i) Tracking of twin nucleation and TB thickening in the Fe-Fe system over time using CSP analysis at different time steps:: (a) 9.5 ps, (b) zoomed area at 9.5 ps, (c) 10 ps, (d) 11 ps, (e) 12 ps, (f) 13 ps, (g) 14 ps, (h) 15 ps, (i) 30 ps; (j) DXA results showing dislocation composition at the TBs; and (k) separation of twinned and untwinned areas by TBs.

In addition to BCC Fe having fewer slip systems, strain rate significantly influences deformation by twinning [71]. Accordingly, when the strain rate exceeds a threshold value, dislocation activity stops, and twins take the responsibility for plastic deformation. Fig. 15 shows twin nucleation and growth mechanisms in the Fe-Fe system. According to Fig. 15a, a twin nucleates at the interface between the crystalline phase and the disordered region towards the crystalline phase.

Fig. 15b represents this twin with the red arrow indicating its growth direction. During the early growth stages, the twin rapidly grows toward its tip until it reaches the particle edge (Figs. 15b-d). Subsequently, after the tip has fully grown, the twin thickening process initiates through growth in lateral directions as the TBs move in the opposite directions (Fig. 15e). As shown in Fig. 15f, this growth may momentarily stop or alter the movement path of TBs, primarily due to the extensive atomic displacement or the intense atomic flow within the particle during severe plastic deformation. However, the twin continues to grow, and as represented in Figs. 15j-i, this results in an increase in twin thickness.

To further clarify the twin growth process, Fig. 15g identifies the Burgers vectors of dislocations situated on the TBs, using DXA analysis. Due to the glide of a perfect ½ [111] dislocation, the twin grows in one direction, leading to the formation of the twin tip. Furthermore, the simultaneous glide of two partial dislocations of 1/3 [111] and 1/6 [111], located at the twin edges, expands and thickens the twin boundaries. Fig. 15k shows the final microstructure of the twin at 30 ps, distinguishing the twinned region from the surrounding areas. The maximum twin thickness was measured to be 1.5 nm.

Deformation twinning in α-Fe, characterized by BCC crystal structure, is a well-documented phenomenon experimentally observed under high strain-rate deformation. In this context, Wang et al. [72] conducted an extensive TEM investigation of α-Fe hollow cylinders subjected to radially propagating shock waves generated by explosive loading. The samples experienced extremely high strain rates (∼10⁶–10⁷ s⁻¹) and significant stress gradients, revealing distinctive twin morphology.

**Fig. 16:**
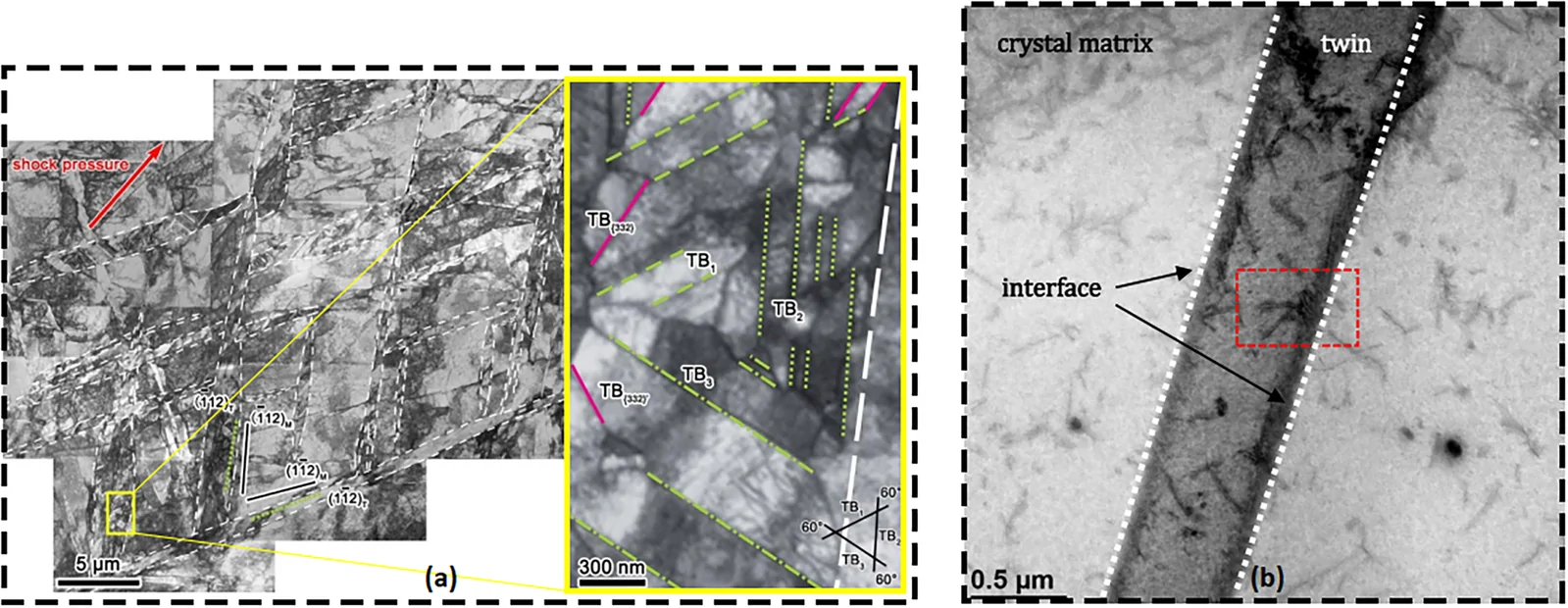
(a) TEM images of a shock-compressed Fe plate, highlighting the area within the yellow rectangle. Three sets of {112} twins are indicated by TB1, TB2, and TB3 (green dashed, dotted, and dash-dot lines), while two sets of {332} twins are marked as T332 and T(332)’ (red solid lines). The black triangle illustrates the angles formed among the three {112} BCC twin systems. (b) Bright-field TEM image of pure Fe following impact, showing a deformation twin. Dislocation arrangements along the twin interface are highlighted in red [72]. All data have been reproduced with permission.

Fig. 16a presents a montage of 18 bright-field TEM images sequentially captured from an area of approximately 35 × 35 μm² within a large α-Fe grain, viewed along the ⟨110⟩ zone axis. The enlarged view of the yellow boxed region in Fig. 16a illustrates three sets of straight edge-on boundaries (marked with green lines), corresponding to intersecting lamellar bands. These TBs represent three sets of (112)⟨111⟩ twins exhibiting threefold symmetry. Additionally, coexisting (332)⟨113⟩ twins are observed alongside the dominant (112)⟨111⟩ system, underscoring the prevalence of deformation twinning in α-Fe microstructures. Ecke et al. [73] conducted an impact experiment in which a hardened steel bolt was accelerated at 50 m/s to impact a cylindrical pure Fe specimen measuring 6 mm in both diameter and height. Fig. 16b presents a bright-field TEM image of the Fe specimen’s cross-section following impact, clearly displaying a deformation twin. Dislocation arrays along the TB (highlighted in red) are responsible for twin propagation within the tip and lateral regions during impact.

Accordingly, a comparison of the microstructural changes observed in the simulations at different impact stages with these experimental results reveals that, first, in BCC-structured Fe under severe plastic deformation regimes, nano-twins are the most significant crystalline defects governing plastic deformation in the material. Furthermore, a critical relationship exists between twin boundaries and twinning dislocations, indicating that the migration and mobility of twin boundaries are inherently dependent on the glide capability of twinning dislocations residing at these boundaries.

**Fig. 17:**
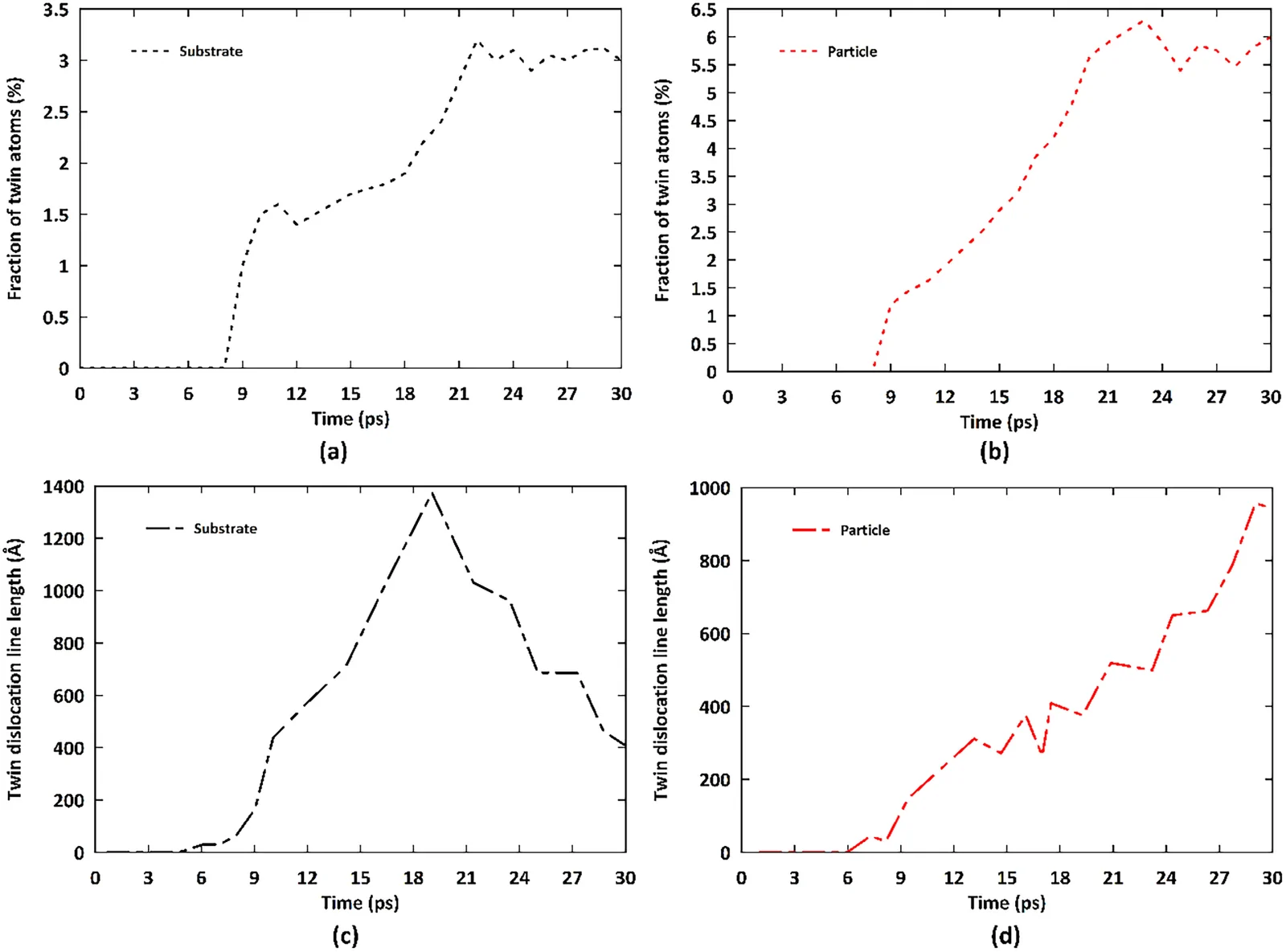
Evolution of deformation twinning during Fe-Fe impact (a,b) Fraction of twin atoms as a function of time in the substrate and particle, respectively (c,d) Corresponding evolution of the total twin-dislocation line length in the substrate and the particle.

To provide a quantitative insight into twinning activity, the fraction of atoms residing at twin boundaries was tracked as a function of time, separately for the particle and the substrate. To this end, similar to [58], surface atoms, amorphous atoms, and grain boundary atoms were computed individually at each time step and subsequently subtracted from the total disordered atom population to approximate the twin boundary atom fraction. As shown in Fig. 17a-b, the twin fraction in the particle consistently exceeds that of the substrate, indicating a higher concentration of twinning-induced plastic deformation within the particle. The progressive increase in twin fraction over time reflects both the continuous nucleation of new twins and the thickening and growth of the previously formed twin lamellae. The twin fraction values obtained for both the particle and the substrate are in good agreement with those reported for pure Fe under shock compression at strain rates of 10⁸ s⁻¹, ranging from 1 to 6% [74]. Moreover, the twin spacing parameter λ observed in the present study falls within 1 to 3 nm, consistent with values reported in previous MD simulations of pure metals deformed at high strain rates (1–4 nm) [22,75].

To further complement this analysis, the total length of twinning dislocations with Burgers vectors of 1/3⟨111⟩ and 1/6⟨111⟩ residing at twin boundaries (Fig. 15j) was computed at successive time steps for both particle and the substrate. The activity of these dislocations in the particle increases continuously throughout all stages of impact (Figs. 17c-d), in good agreement with the twin fraction trend observed in Fig. 17a-b, confirming that twinning activity is more pronounced and sustained in the particle than in the substrate.

### 3.5 BCC system: dislocation-assisted twinning

**Fig. 18:**
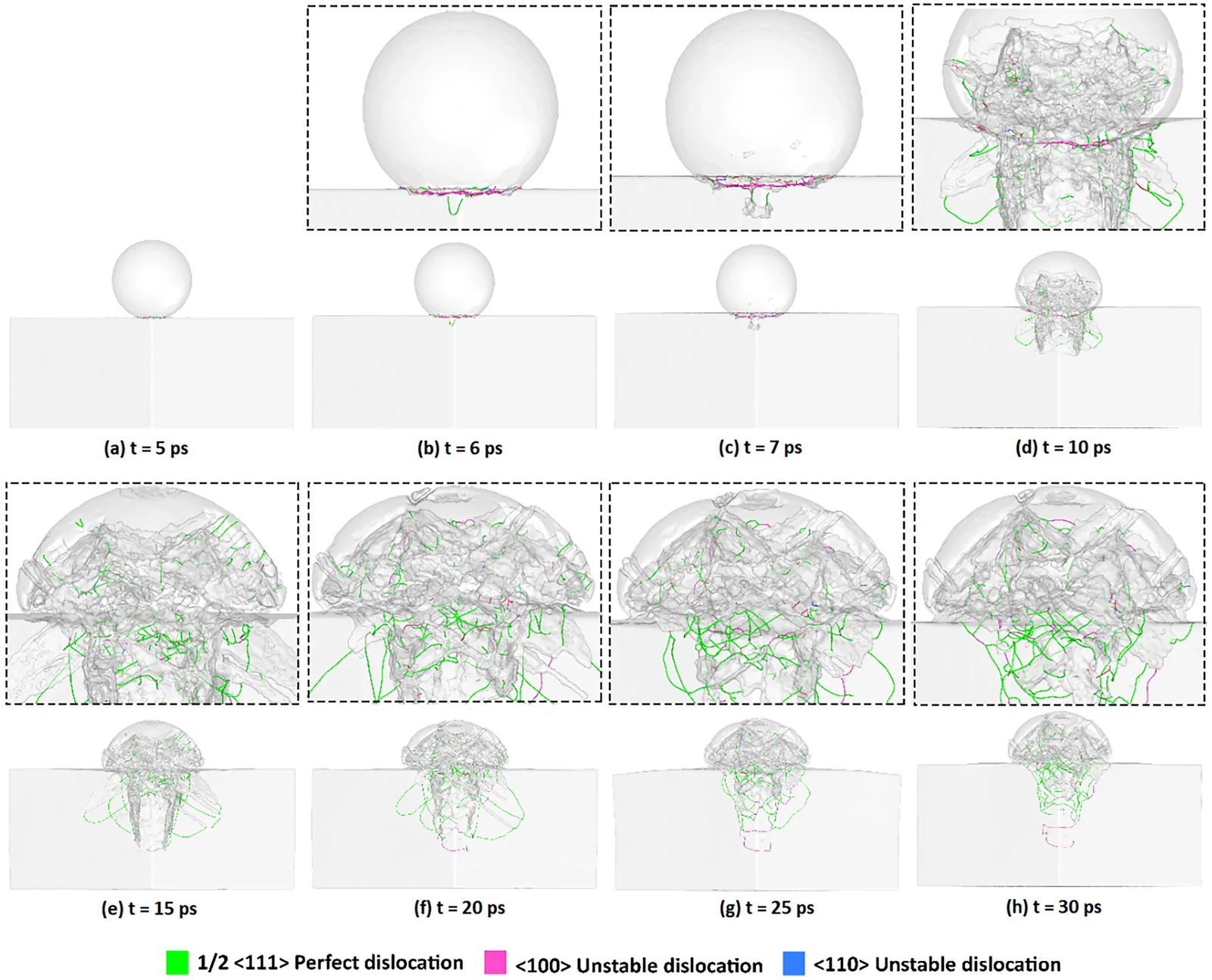
Qualitative DXA analysis of the Fe–Fe system showing the evolution of dislocation lines over time: (a) 5 ps, (b) 6 ps, (c) 7 ps, (d) 10 ps, (e) 15 ps, (f) 20 ps, (g) 25 ps, and (h) 30 ps.

Twinning serves as the primary mechanism for plastic deformation in both Fe particle and the substrate. Consequently, it is anticipated that perfect dislocations, essential for initiating and propagating twins, will be observed in significant quantities in the system, as indicated in Fig. 18, which illustrates the dislocations formed at various stages of impact.

As depicted in Figs. 18a-d, during the initial instants of impact, dislocations are concentrated at the interface and gradually occupy the entire contact zone between the particle and the substrate. These dislocations can be categorized into three groups: ½<111>, 〈110〉, and 〈100〉. The first group consists of perfect dislocations, while the latter two are classified as unstable and partial dislocations. The formation of such partial dislocations in the BCC structure of Fe is rare and plays a minimal role in plastic deformation. As shown in Fig. 18, during the intermediate and final stages of impact (15–30 ps), perfect dislocations migrate from the interface to the central regions of both particle and the substrate. Considering the twins illustrated in Figs. 11e–h, these dislocations are expected to serve as critical agents in twin formation within the Impact-Affected Zone (IAZ) of both components, which denotes the near-impact region where severe plastic deformation and high strain rates lead to pronounced microstructural evolution (e.g., dislocation accumulation and twinning).

**Fig. 19:**
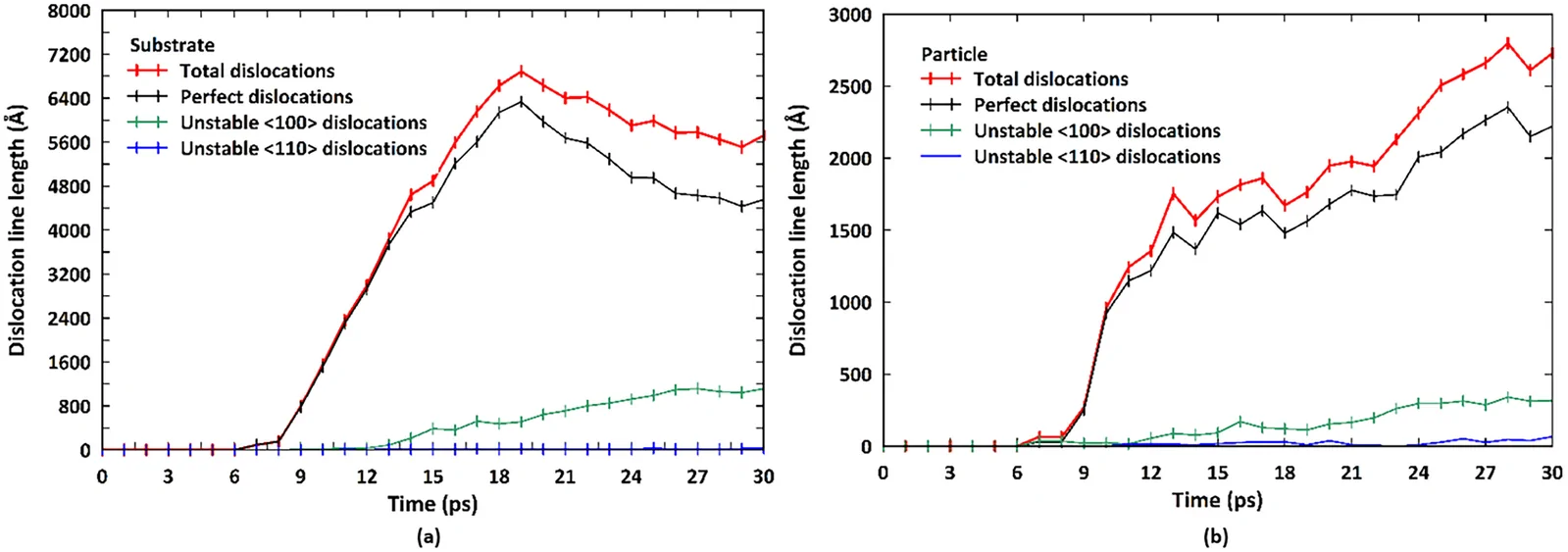
Quantitative DXA analysis of the Fe–Fe system showing the evolution of dislocation line length over time: (a) dislocations in the substrate, and (b) dislocations in the particle.

A comparison of Fig. 7, Fig. 18 reveals that the dense, entangled dislocation forest observed in the FCC Al-Al system is absent in the BCC Fe-Fe microstructure. This contrast is further underscored by a quantitative analysis of the total lengths of dislocation lines generated in the BCC system (Fig. 19) compared to the FCC counterpart (Fig. 9). In the BCC system, the total dislocation line lengths reach approximately 7000 Å in the substrate and 2700 Å in the particle, whereas in Al-Al system, these lengths are 55,000 Å and 12,000 Å, respectively. Additionally, these differences can be attributed not only to the nature of the crystal structure but also to the stacking fault energy (SFE) of each material. SFE of Al (160 mJ/m²) is considerably lower than that of BCC-structured Fe. Since a lower SFE promotes greater stability of stacking fault planes and the stacking faults in FCC metals typically form between two Shockley partial dislocations, this results in a substantially higher density of partial dislocations in Al, further contributing to the observed disparity in dislocation density between the two systems. This demonstrates that, under similar impact velocities, the extent of plastic deformation is significantly greater in the FCC system compared to the BCC one. A trend similar to the FCC system is noted here in the later stages of impact, wherein dislocation density decreases. However, in the BCC system, the total dislocation density in the Fe particle, including perfect dislocations, increases over time. This behavior can be primarily attributed to the activation of twinning during the intermediate and later stages, which enhances dislocation glide through twin growth, resulting in longer dislocation lines. An interesting reversal, however, emerges when tracking the evolution of dislocation density between 20 ps and 30 ps: while the substrate experiences a gradual decline, the particle undergoes a marked rise in dislocation activity. This shift reflects a redistribution of plastic deformation between the two bodies in the later stages of impact. As kinetic energy progressively dissipates, the substrate's resistance to further penetration grows, and solidification of the molten material at the interface restricts additional particle ingress. Under these conditions, impact-induced stress waves redirect plastic deformation back into the particle, fueling intense dislocation activity that ultimately governs the morphological evolution of the particle and its departure from the initial spherical geometry.

### 3.6 FCC and BCC CS systems: recrystallization/recovery

**Fig. 20:**
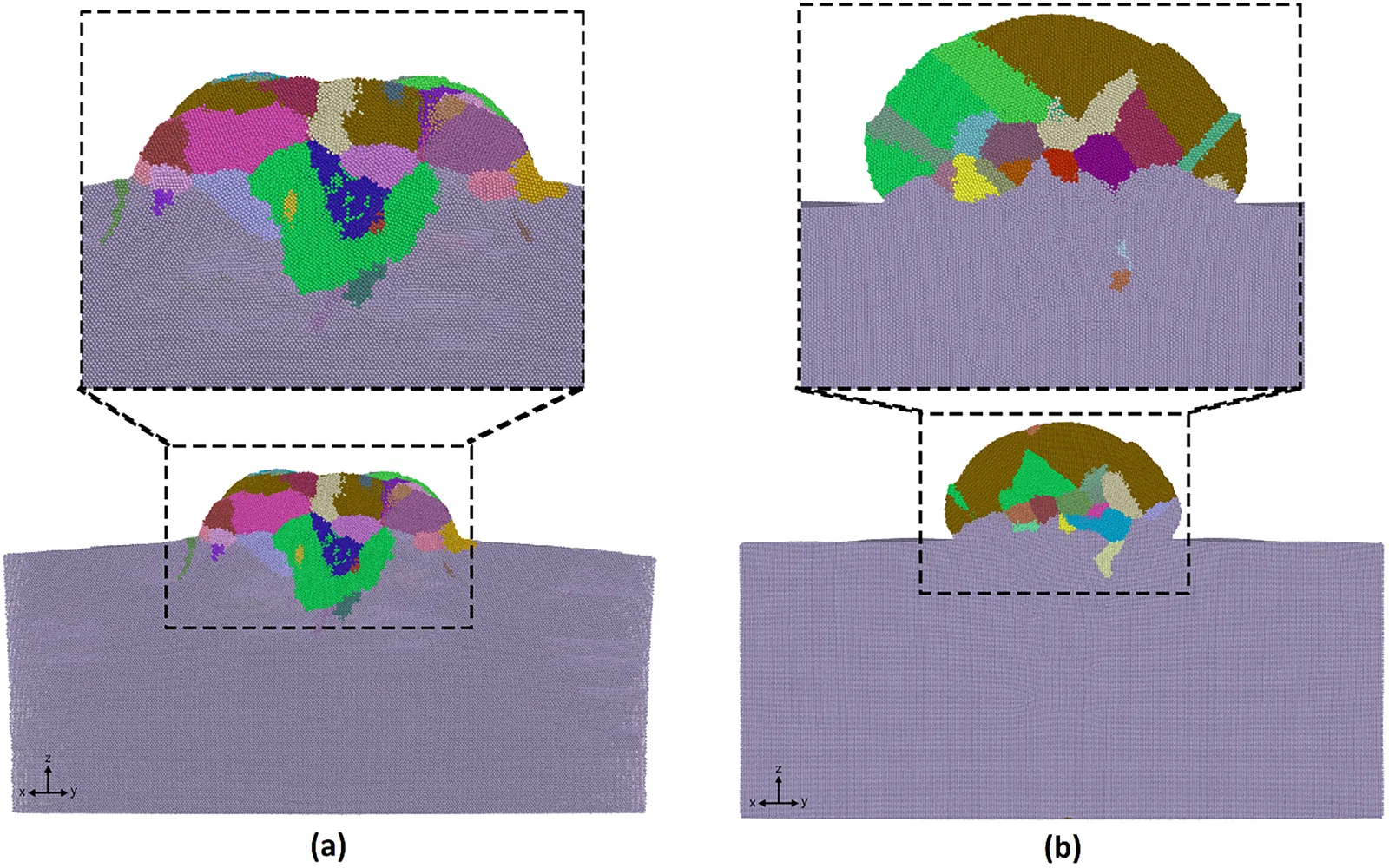
Grain segmentation analysis of the final (at 30 ps) microstructure for: (a) Al-Al FCC system, and (b) Fe-Fe BCC system.

According to the ASI theory, the heat generation rate due to particle impact in CS exceeds the rate of thermal diffusion [44]. Consequently, heat persists within the interface region, potentially softening the material locally [44]. This softening assists in jetting and breaking oxide layers at the interface, while also promoting the integration of particle atoms into the substrate [76]. Furthermore, the retention of thermal energy in the interface and surrounding IAZ appears to activate additional thermally driven mechanisms. In particular, this thermal energy can enable dislocation recovery, eliminate some crystal defects while reorganizing others into subgrains, as discussed in 3.1, 3.3. Dynamic recrystallization, thus, becomes a key phenomenon, producing ultra-fine, strain-free grains that enhance bonding strength. Fig. 20 compares the final microstructures of Al-Al and Fe-Fe systems at 30 ps based on grain segmentation analysis. Figs. 20a and 20b evidence that in both systems recrystallization-induced grain formation is more pronounced within the particle than the substrate, indicating that a considerable portion of particle impact energy is dissipated through plastic deformation in the particle when impacting a substrate of similar material.

**Fig. 21:**
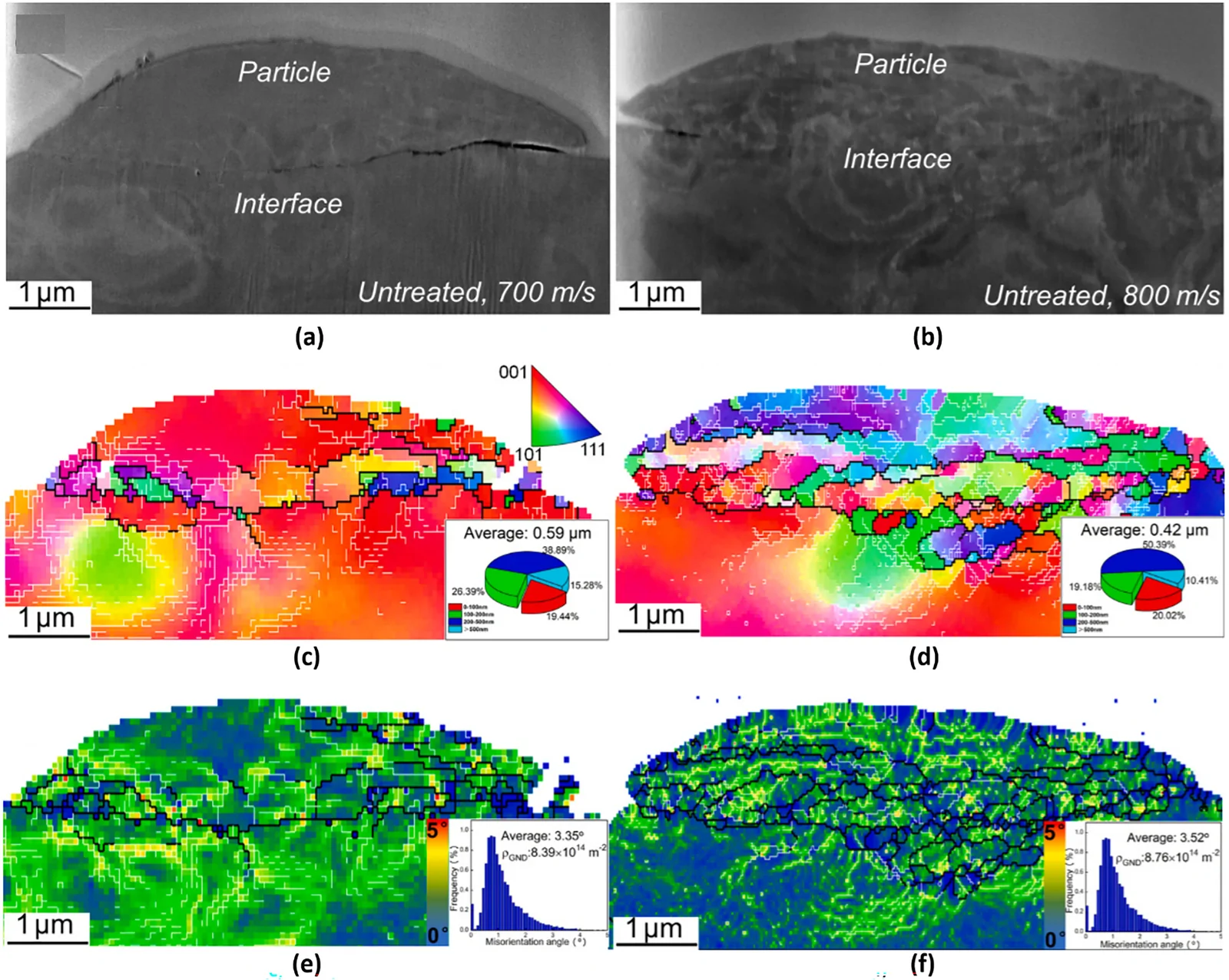
(a-b) SEM micrograph of an Al particle deposited on Al substrate: (a) impact velocity of 750 m/s, and (b) 800 m/s. (c-d) Inverse Pole Figure (IPF) maps: (c) 750 m/s, (d) 800 m/s. (e-f) the Kernel Average Misorientation (KAM) and GND density maps: (c) 750 m/s, and (f) 800 m/s [77]. All data have been reproduced with permission.

Figs. 21a-b show SEM micrographs of CS deposited Al particle on an Al substrate at impact velocities of 700 and 800 m/s, respectively [77]. The distribution of recrystallized grains for these samples presented in Figs. 21c-d indicates that the newly formed grains during recrystallization are concentrated in the interfacial region between particle and the substrate, which strongly supports our simulation results presented in Fig. 20. Furthermore, the dislocation density maps obtained from Geometrically Necessary Dislocation (GND) analysis, as shown in Figs. 21e-f, also confirm the dislocation forest structure observed in the Al-Al system presented in Fig. 7. A more quantitative perspective can be gained by normalizing the total dislocation line length reported in Fig. 9 by the total system volume, yielding an estimated dislocation density of approximately 6.58 × 10¹⁶ m⁻² at the final simulation time step. While this value exceeds the GND density of approximately 8.76 × 10¹⁴ m⁻² extracted from the experimental results of Fig. 21f, it nonetheless falls within the range commonly reported for metallic systems undergoing severe plastic deformation in MD simulations. The difference can be largely accounted for by the higher impact velocity used in the present study (1200 m/s versus 800 m/s in the experimental reference), the characteristically elevated strain rates inherent to MD frameworks, as well as the spatiotemporal constraints of MD models that limit dislocation recovery and relaxation processes.

The comparison with the experimental results presented in Fig. 21 is primarily qualitative in nature, as the impact velocity employed in the present simulation (1200 m/s) exceeds the velocity range reported in the reference experimental study (approximately 700–800 m/s). This discrepancy arises from the inherent length-scale limitations of MD simulations, where particle dimensions are several orders of magnitude smaller than those used in real cold spray process. At the nanoscale, higher impact velocities are generally required to induce sufficient plastic deformation and achieve interfacial bonding, as the critical bonding velocity increases with decreasing the particle size. Despite this difference, the similarities in deformation mechanisms and microstructural features, including fine grain formation in the interfacial region, dislocation accumulation, and the final morphology of the impact zone, indicate reasonable qualitative agreement between the simulation results and experimental observations. The purpose of this comparison is therefore to provide a qualitative validation of the microstructural trends and physical mechanisms governing the impact process.

**Fig. 22:**
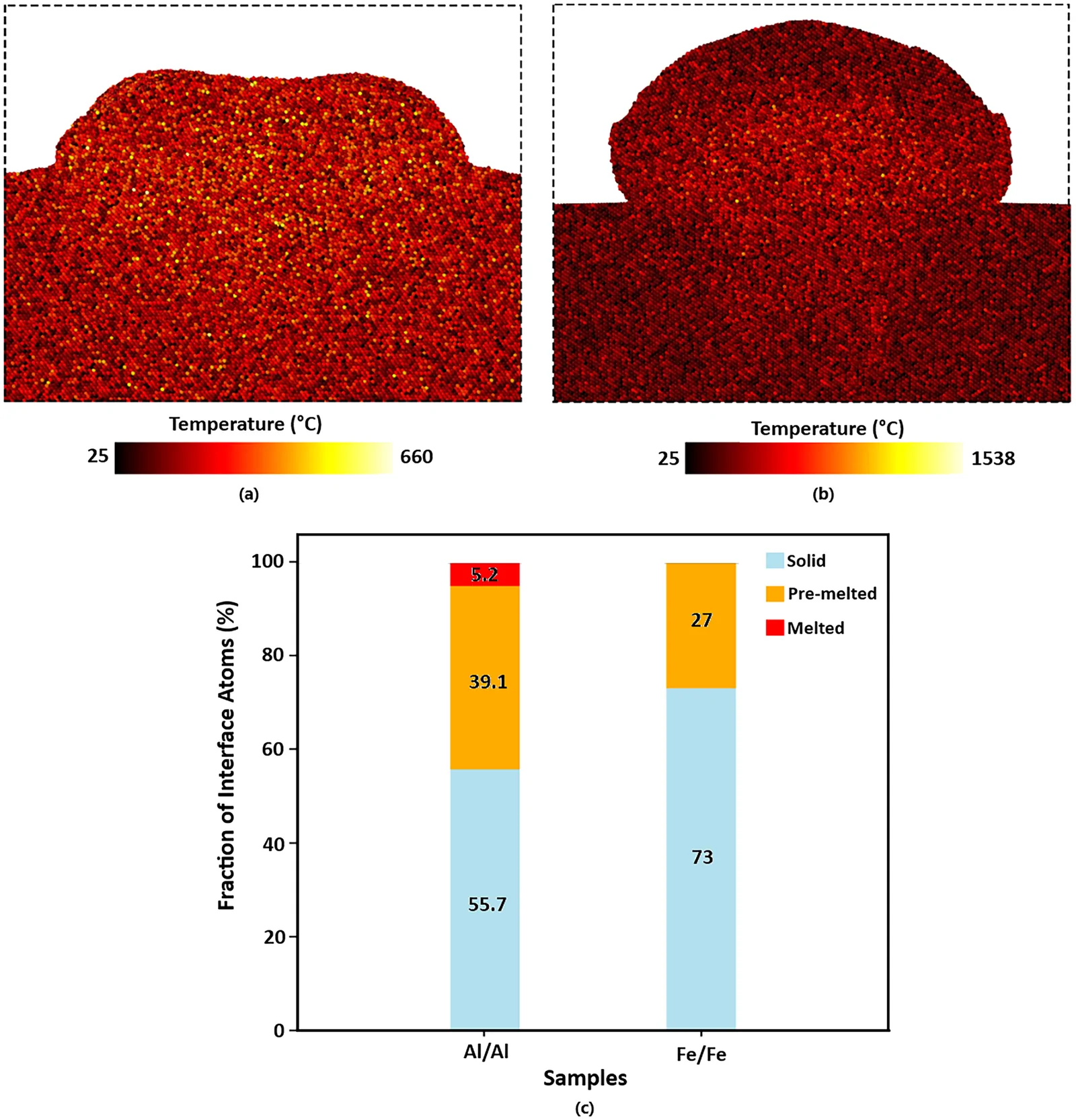
(a-b) Temperature profile of both FCC and BCC systems at 20 ps. (a) Al-Al, and (b) Fe-Fe. (c) Quantitative temperature distribution in both FCC and BCC systems.

Fig. 20 illustrates a distinct contrast in the interface region of the two systems: the newly formed grains in the FCC system are substantially larger (approximately 7.5 nm) than the finer grains present in the BCC system (approximately 1.6 nm). This difference could likely be due to greater heat generation in Al-Al, attributable to Al’s lower melting point compared to Fe (T_mAl_ ∼660 °C vs. T_mFe_ ∼1538 °C). Fig. 22 illustrates the temperature distribution at 20 ps, representing the intermediate impact stage, for both FCC and BCC systems. In this figure, the pre-melting temperature is considered around 100 °C below the nominal melting point. Comparing Figs. 22a and 22b shows that, at a similar temperature, impact velocity, and geometry, a larger fraction of interface atoms in the FCC system remains at pre-melting temperatures. Fig. 22C shows that, quantitatively, around 5.2% of the interface atoms in the FCC system remain at their melting temperature, whereas the BCC system has mostly cooled down and is progressing through the final solidification stage. These observations suggest that thermal energy generated during impact is retained longer in the FCC system than in the BCC one, which likely promotes grain growth and results in larger grains at the corresponding interface region. The larger grain size observed in Al relative to Fe cannot be attributed solely to greater thermal stability. Beyond thermal effects, this difference may also stem from distinct recovery behavior and recrystallization kinetics in the two materials. Recrystallization processes are strongly governed by the homologous temperature (T/Tm), where Tm denotes the melting point of the material. Since the melting point of Al is considerably lower than that of Fe, Al experiences a significantly higher homologous temperature under identical impact conditions [78]. Such conditions are more conducive to the activation of dynamic recovery, recrystallization, and grain growth in the FCC system, thereby facilitating the formation of larger grains in the interfacial region. Furthermore, the FCC crystal structure of Al promotes dislocation rearrangement and cross-slip during severe impact-induced deformation, whereas Fe under high-strain-rate conditions tends to retain a higher defect density and greater stored deformation energy, leading to a more pronounced sub-grain subdivision and the formation of finer microstructures. Although differences in density and impact-induced energy partitioning may influence the local thermomechanical response of the materials, the higher homologous temperature of Al, together with its enhanced dynamic recovery and recrystallization kinetics associated with the FCC crystal structure, are expected to be the primary factors promoting grain coarsening. Consequently, the larger grain size observed in Al is most likely the result of the combined effects of its higher homologous temperature and more efficient recovery and recrystallization mechanisms relative to Fe.

## 4 Conclusion

The primary objective of this study has been to clarify how severe plastic deformation and inherent material properties influence the bonding state in CS systems composed of powder and substrates of similar nature. To accomplish this objective, MD simulations were developed to analyze the particle impact process for FCC (Al-Al) and BCC (Fe-Fe) systems at atomic level. The findings can be summarized as follows:

During the initial instants of impact, von-Mises stress distribution analysis indicated stress concentration mainly within the interface between the particle and the substrate, which triggered microstructural changes for both systems. The high impact energy induced significant plastic deformation not only at the interface, but also within the particle and a wider region of the substrate, named as impact affected zone. Notably, in both BCC and FCC systems, plastic deformation was more pronounced in the particle than in the substrate, although the dominant deformation mechanisms differed between the two cases.

According to PTM analysis, Al underwent a solid-to-solid phase transformation involving a transition from FCC-to-BCC and FCC-to-HCP structures. Nonetheless, the Al atoms within the BCC structure were transient and later reverted to the FCC structure. Additionally, melting in the rim region at the interface facilitated further diffusion of particle atoms into the substrate. In contrast, for Fe, plastic deformation occurred through BCC phase transformation to disordered atoms, accompanied by significant twin formation.

During the intermediate impact stage, DXA analysis revealed a substantial increase in dislocation density in Al compared to Fe. The results indicate that a considerable portion of these dislocations are of the Shockley partial type, formed via direct nucleation and decomposition of perfect dislocations within the system. The interactions among these dislocations led to the formation of locked dislocations, such as Hirth 1/3<$\bar{1}$00> and Stair-rod 1/6 < 1$\bar{1}$0>, which inhibit the slip of other dislocations, thereby enhancing bonding at the particle-substrate interface. As regards the Fe-Fe system, DXA analysis indicated perfect dislocations to be predominant. Furthermore, a significant correlation was observed between perfect dislocations in the BCC lattice and twin formation. Perfect dislocations with a Burgers vector of 1/2[111] were associated with twin tips, while two types of partial dislocations, i.e. 1/3[111] and 1/6[111], were found at twin boundaries. Partial dislocation glide caused twin thickening, while perfect dislocation glide promoted twin growth. While twin formation and thickening promote plastic deformation, they can also impede dislocation slip by changing slip systems, thereby promote local bonding.

At the final stage of impact, grain segmentation analysis indicated that both systems underwent recrystallization leading to the formation of new grains in both particle and substrate, predominantly within the particle. This behavior is attributed to the severe shear strain and thermal recovery of dislocations due to heat retention within the impacted area. A comparison of the newly formed grain distribution maps revealed that the grains formed at the interface in Al-Al system were nearly five times larger than those in the Fe-Fe system (7.5 nm vs 1.6 nm). This disparity can be attributed to the greater thermal stability of the heat generated during impact in the FCC system compared to the BCC one, which intensifies grain coarsening and subsequently increases grain size at the interface region. Regardless of grain size, the emergence of newly formed grains, which were significantly finer than the original grains of both the powder and substrate, can enhance bonding strength at the interface by increasing the number of GBs, which serve as obstacles to dislocation slip paths.

The results from the atomic-scale MD simulations presented in this study reveal key microstructural phenomena occurring at various stages of impact in similar CS systems of FCC and BCC nature and clarify the difference in the fundamental mechanisms responsible for bond strengthening that can be contributed to the inherent structural properties of the materials.

## Funding sources

The authors acknowledge ERC-CoG ArcHIDep funding from the European Research Council (ERC) under grant agreement n. 101044228. Views and opinions expressed are, however, those of the authors only and do not necessarily reflect those of the European Union or the European Research Council Executive Agency. Neither the European Union nor the granting authority can be held responsible for them.

## Data availability

All data are available in the main text or the supplementary materials.

## CRediT authorship contribution statement

**Arash Kardani:** Writing – original draft, Validation, Software, Methodology, Investigation, Formal analysis, Data curation, Conceptualization. **Sara Bagherifard:** Writing – review & editing, Supervision, Resources, Funding acquisition, Formal analysis, Conceptualization.

## Declaration of competing interest

The authors declare that they have no known competing financial interests or personal relationships that could have appeared to influence the work reported in this paper.

## Data Availability

All data are available in the main text or the supplementary materials.
